# Genetic correlates of vitamin D-binding protein and 25-hydroxyvitamin D in neonatal dried blood spots

**DOI:** 10.1038/s41467-023-36392-5

**Published:** 2023-02-15

**Authors:** Clara Albiñana, Zhihong Zhu, Nis Borbye-Lorenzen, Sanne Grundvad Boelt, Arieh S. Cohen, Kristin Skogstrand, Naomi R. Wray, Joana A. Revez, Florian Privé, Liselotte V. Petersen, Cynthia M. Bulik, Oleguer Plana-Ripoll, Katherine L. Musliner, Esben Agerbo, Anders D. Børglum, David M. Hougaard, Merete Nordentoft, Thomas Werge, Preben Bo Mortensen, Bjarni J. Vilhjálmsson, John J. McGrath

**Affiliations:** 1 National Centre for Register-Based Research, Aarhus University, 8210 Aarhus V, Denmark; 2 Center for Neonatal Screening, Department of Congenital Disorders, Statens Serum Institut, Copenhagen, Denmark; 3 Clinical Mass Spectrometry, Statens Serum Institut, Artillerivej 5, DK-2300 Copenhagen S, Denmark; 4 Testcenter Denmark, Statens Serum Institut, Artillerivej 5, DK-2300 Copenhagen S, Denmark; 5 Institute for Molecular Bioscience, University of Queensland, Brisbane, QLD Australia; 6 Queensland Brain Institute, University of Queensland, Brisbane, QLD Australia; 7 The Lundbeck Foundation Initiative for Integrative Psychiatric Research, iPSYCH, 8210 Aarhus V, Denmark; 8 Department of Psychiatry, University of North Carolina at Chapel Hill, Chapel Hill, NC USA; 9 Department of Medical Epidemiology and Biostatistics, Karolinska Institutet, Stockholm, Sweden; 10 Department of Nutrition, University of North Carolina at Chapel Hill, Chapel Hill, NC USA; 11 Department of Clinical Epidemiology, Aarhus University and Aarhus University Hospital, 8200 Aarhus N, Denmark; 12 Department of Affective Disorders, Aarhus University and Aarhus University Hospital-Psychiatry, Aarhus, Denmark; 13 CIRRAU - Centre for Integrated Register-based Research, Aarhus University, Aarhus, Denmark; 14 Center for Genomics and Personalized Medicine, Aarhus University, Aarhus, Denmark; 15 Department of Biomedicine and the iSEQ Center, Aarhus University, Aarhus, Denmark; 16 Department for Congenital Disorders, Statens Serum Institut, 2300 Copenhagen S, Denmark; 17 Mental Health Services in the Capital Region of Denmark, Mental Health Center Copenhagen, University of Copenhagen, 2100 Copenhagen, Denmark; 18 Department of Clinical Medicine, University of Copenhagen, 2200 Copenhagen N, Denmark; 19 Institute of Biological Psychiatry, Mental Health Services, Copenhagen University Hospital, Copenhagen N, Denmark; 20 Department of Clinical Medicine, University of Copenhagen, Copenhagen, Denmark; 21 Lundbeck Center for Geogenetics, GLOBE Institute, University of Copenhagen, Copenhagen, Denmark; 22 Bioinformatics Research Centre, Aarhus University, 8000 Aarhus C, Denmark; 23 Queensland Centre for Mental Health Research, The Park Centre for Mental Health, Brisbane, QLD 4076 Australia

**Keywords:** Genetics research, Epidemiology

## Abstract

The vitamin D binding protein (DBP), encoded by the group-specific component (*GC*) gene, is a component of the vitamin D system. In a genome-wide association study of DBP concentration in 65,589 neonates we identify 26 independent loci, 17 of which are in or close to the *GC* gene, with fine-mapping identifying 2 missense variants on chromosomes 12 and 17 (within *SH2B3* and *GSDMA*, respectively). When adjusted for *GC* haplotypes, we find 15 independent loci distributed over 10 chromosomes. Mendelian randomization analyses identify a unidirectional effect of higher DBP concentration and (a) higher 25-hydroxyvitamin D concentration, and (b) a reduced risk of multiple sclerosis and rheumatoid arthritis. A phenome-wide association study confirms that higher DBP concentration is associated with a reduced risk of vitamin D deficiency. Our findings provide valuable insights into the influence of DBP on vitamin D status and a range of health outcomes.

## Abstract (web summary)

In this study the authors measure the concentration of 25-hydroxyvitamin D and vitamin D binding protein (DBP) in 65,589 neonatal dried blood samples. Findings from further analyses include that the genetic correlates of DBP concentration predict the risk of vitamin D deficiency.

## Introduction

The vitamin D binding protein (DBP) is a highly polymorphic protein best known for its role related to the transport of 25-hydroxyvitamin D (25OHD) and 1,25-dihydroxvitamin D (1,25OHD)^1^. DBP, which is an abundant circulating (plasma) protein structurally related to albumin, is encoded by the group-specific component (*GC*) gene. Haplotypes determined by two missense variants in the *GC* gene (rs7041 and rs4588) determine key isoforms of the DBP protein, which are labeled according to their electrophoretic properties (1S, 1F, 2). Apart from these haplotypes, there are many additional variants in humans^1^.

While DBP also has a range of additional roles (e.g., actin scavenging after tissue injury, C5a-mediated chemotaxis, T-cell response, macrophage activation^2^), most research has focused on the contribution of DBP to overall vitamin D status. It is known from related steroid hormones and their binding proteins, that the concentration of the binding protein can influence the bioavailability of the target hormone. Much of this research has been informed by the free hormone hypothesis^3^, which proposes that the biological activity of a hormone is related to the unbound (i.e., free) rather than the protein-bound concentration in the plasma^1^. With respect to the total 25OHD, on average only 0.03% is free, 85% is bound to DBP, and the remainder is bound (less strongly) to albumin^4^. Apart from some tissues which can retrieve protein-bound 25OHD via endocytosis (e.g., distal renal tubules, the placenta), free/unbound 25OHD is thought to be the biologically active fraction. Recently, an individual with a homozygous deletion of *GC* was identified, and shown to have DBP and 25OHD concentrations below the limit of detection (the concentration of 1,25OHD was low but detectable)^5^. Interestingly, the affected individual did not display adverse bone outcomes traditionally associated with vitamin D deficiency. Combined with evidence from transgenic animal models of *GC* knock-outs^6^, this indicates that DBP is not necessary for the transport of 25OHD throughout the body, nor is DBP necessary for general bone health. It is now appreciated that the concentration of DBP can influence the half-life of 25OHD^5,7^. When the concentration of DBP is lower, then more 25OHD is free/unbound, and this fraction of the total 25OHD is more rapidly transferred to target cells and subsequently catabolized, thus shortening the functional half-life of 25OHD.

Despite concerns about the accuracy of some DBP assays (assays based on monoclonal antibodies have underestimated DBP concentration in African-Americans^8^), it is clear that there is appreciable variation in DBP concentration within groups sorted by DBP isoform type^8^. Some of this variation may be related to genetic factors. To date, we are aware of only one genome-wide association study (GWAS) of DBP concentration, based on 1380 men^9^. This study, which used a monoclonal antibody, identified two genome-wide significant loci, both of which were within the *GC* gene (rs7041 and rs705117). Of particular interest, variants in *GC* are also strongly and consistently associated with the concentration of 25OHD^10–13^, indicating a critical role for the binding protein in predicting the total 25OHD concentration. In light of the importance of DBP concentration for influencing vitamin D status, there is a need to undertake GWAS analyses of DBP concentration in larger samples, and to explore analytic methods that can take into account potential isoform-specific assay biases. The summary statistics from this type of study could then be used in a range of Mendelian randomization studies (in particular, the association between DBP concentration and 25OHD concentration) and phenome-wide association studies (PheWAS). While many disorders have been linked to vitamin D-related pathways^14–16^, in the Mendelian randomization studies we will focus on (a) a set of neurological, psychiatric and cognitive phenotypes that have been linked to vitamin D pathways (schizophrenia^17,18^, major depression^19^, bipolar disorder^20^, ASD^21–25^, ADHD^26,27^, Alzheimer’s disease^28,29^, amyotrophic lateral sclerosis^30^, educational attainment^11^) and (b) selected autoimmune disorders also linked to vitamin D pathways (multiple sclerosis^31,32^, type 1 diabetes [T1D]^33,34^, Crohn’s disease^35^, ulcerative colitis^35^, and rheumatoid arthritis^36^). We will explore a much wider range of outcomes in the PheWAS (1027 disease phenotypes).

**Fig. 1:**
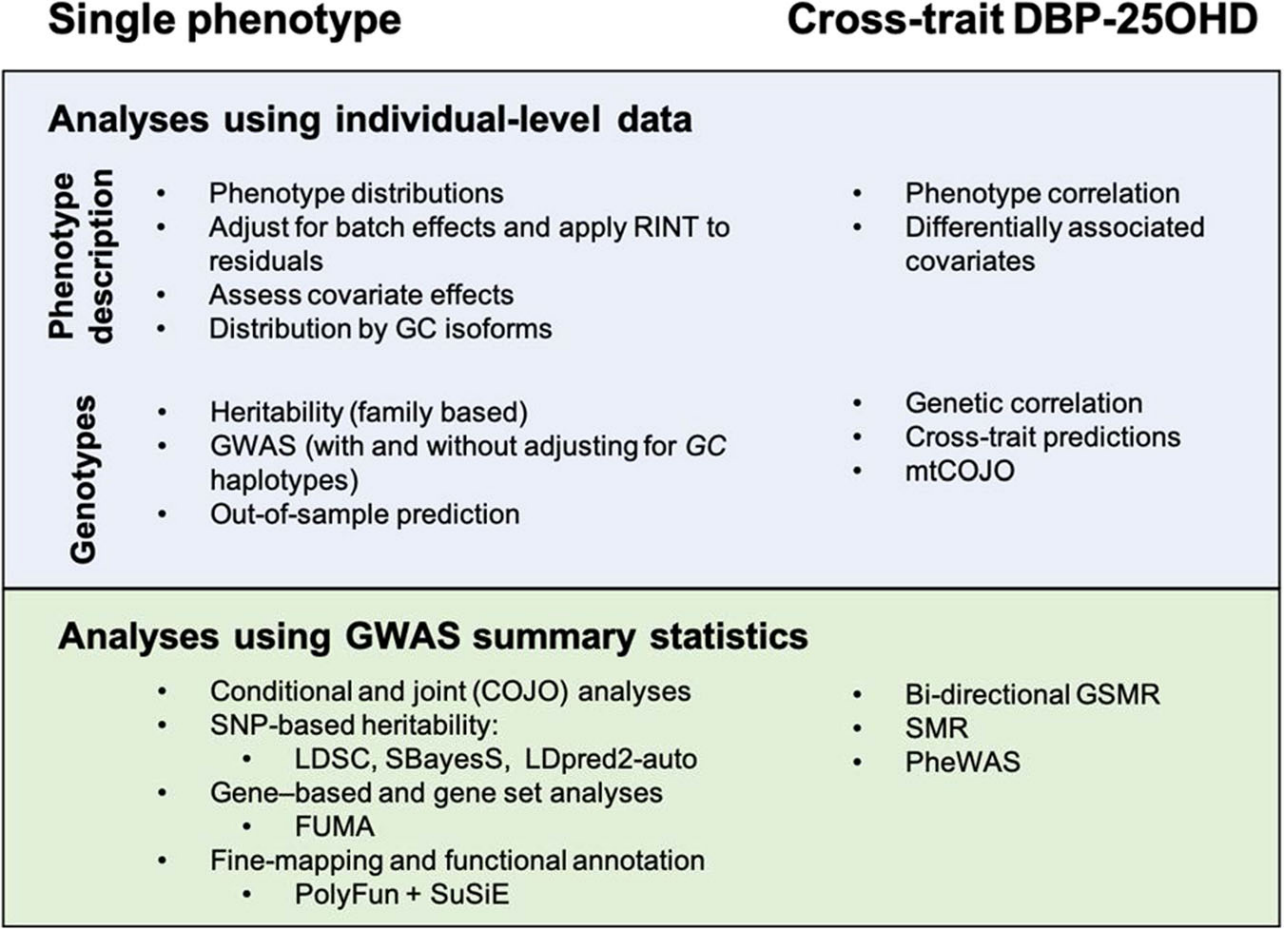
Methods figure. FUMA functional mapping and annotation of genome-wide association studies, SMR summary-data–based Mendelian randomization, GSMR generalized summary-data-based Mendelian randomization, PheWAS phenome-wide association study, SNP single-nucleotide polymorphism, LDSC LD-score regression, mtCOJO multi-trait-based conditional and joint association analysis using GWAS summary statistics, SuSiE “sum of single effects”.

In this work, we measure both DBP and 25OHD concentrations in a large sample of neonatal dried blood spots^37^. Because these samples have previously been genotyped, we were able to undertake a GWAS of DBP (Fig. 1). The aims of this study are to (a) describe the distribution and epidemiological correlates of DBP in neonatal dried blood spots, (b) examine single-nucleotide polymorphism (SNP)-based and family-based heritability of DBP, and (c) undertake a GWAS of DBP. Based on the results of the GWAS, we (d) use bioinformatics tools to explore properties of the genome-wide significant loci, (e) use Mendelian randomization to explore the association between DBP and 25OHD concentration, and between DBP and a range of potential vitamin D-related candidate disorders and traits (including neuropsychiatric and autoimmune-related disorders), and (f) conduct a phenome-wide association study (PheWAS)^38^ to examine the relationship between the genetic correlates of DBP and a wide range of health phenotypes.

## Results

### 25OHD and DBP phenotypes

Of the 71,944 and 71,212 individuals who had DBP and 25OHD neonatal blood concentrations respectively, 65,694 had data on both. Distributions of both measures were right-skewed (Supplementary Fig. 6) with a Pearson’s correlation coefficient between them of 0.19 (*P* value <2.2 × 10^−16^) (Supplementary Fig. 7). As expected, 25OHD concentration showed prominent seasonal fluctuation, but there was no seasonal fluctuation in DBP concentrations (Supplementary Fig. 8). Based on the sample used in the GWAS study, the mean, median, standard deviation and interquartile range of 25OHD were 23.66, 22.12, 14.06, 14.04–145.19 nmol/L, respectively. These values, which are lower than concentrations generally found in adult samples^39^, are consistent with previous Danish studies of 25OHD based on neonatal dried blood spots^32,40^.

**Fig. 2:**
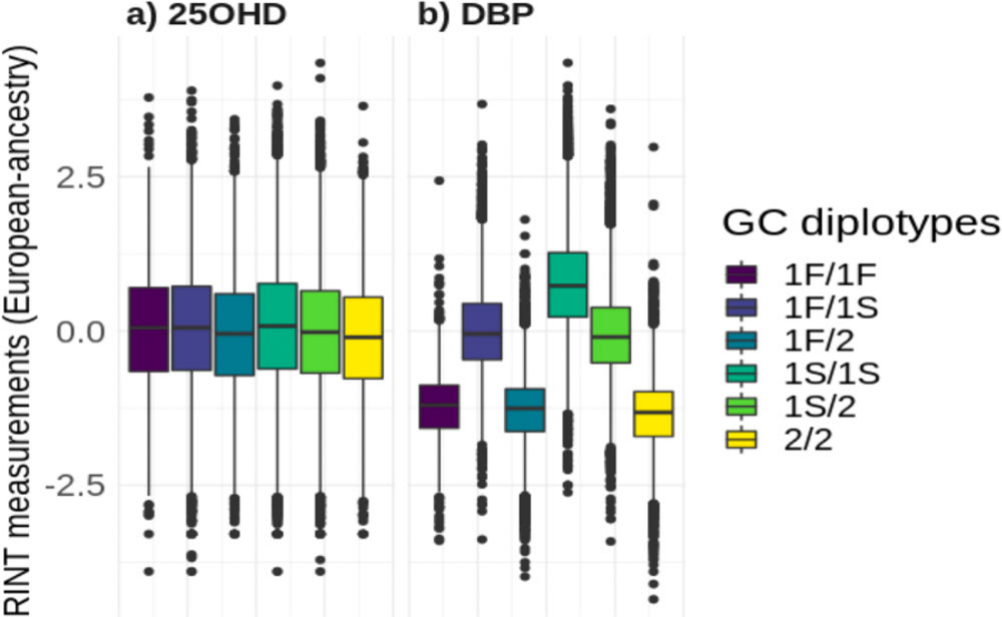
Distributions of transformed 25-hydroxyvitamin D (25OHD) and vitamin D binding protein (DBP) by the six diplotypes, within the European ancestry subgroup. The colors represent the six diplotype combinations. The center lines show the medians; box limits indicate the 25th and 75th percentiles; whiskers extend 1.5 times the interquartile range from the 25th and 75th percentiles; outliers are represented by dots. The sample sizes for the 1 F/1 F, 1 F/1 S, 1 F/2, 1 S/1 S, 1 S/2 NS 2/2 diplotypes for **a** 25OHD are 1452, 10,986, 5262, 21,694, 20,644, and 4950 respectively; and **b** DBP are 1501, 11,077, 5314, 21,883, 20,801, and 5013 respectively.

Three main haplotypes were inferred from the two well-characterized loci within the *GC* gene (rs7041 and rs4588; Supplementary Tables 1, 2)^1^. The distributions of DBP and 25OHD for each of the six possible haplotype combinations (i.e., diplotypes reflecting the contribution of the different haplotypes on each chromosome) for the European ancestry subsample is shown in Fig. 2 (the same figure for the entire sample can be found in the Supplementary Fig. 9).

The six *GC* diplotypes were significantly associated with both DBP and 25OHD levels in the entire sample and European ancestry subsample (ANOVA *P* value <2 × 10^−16^; Fig. 2 and Supplementary Fig. 9). In keeping with previous literature based on monoclonal antibodies^8^, the 1 S *GC* isoform was associated with higher DBP concentrations. The proportion of DBP variance explained by the *GC* haplotypes was 52.6%, while it was only 0.8% for the 25OHD levels. For completeness, we also show the relative proportion of *GC* haplotypes in the non-European sample. As expected, the 1 F isoform was more prevalent in those with African ancestry (Supplementary Fig. 10).

To estimate the heritability of 25OHD and DBP, we used GCTA-GREML^41^, and obtained SNP-heritability estimates from GREML, SBayesS, and LDpred2-auto. The last two can model sparse genetic architectures, while GREML assumes an infinitesimal architecture. For the family-based heritability estimate, we used a set of 6313 related individuals with a coefficient of relationship (r) >0.2 to at least one other person in the set (all relatives). For 25OHD, the family-based heritability (and standard error) was 0.36 (0.03) and 0.35 (0.03) after adjustment for GC haplotypes (Supplementary Data 2).

**Fig. 3:**
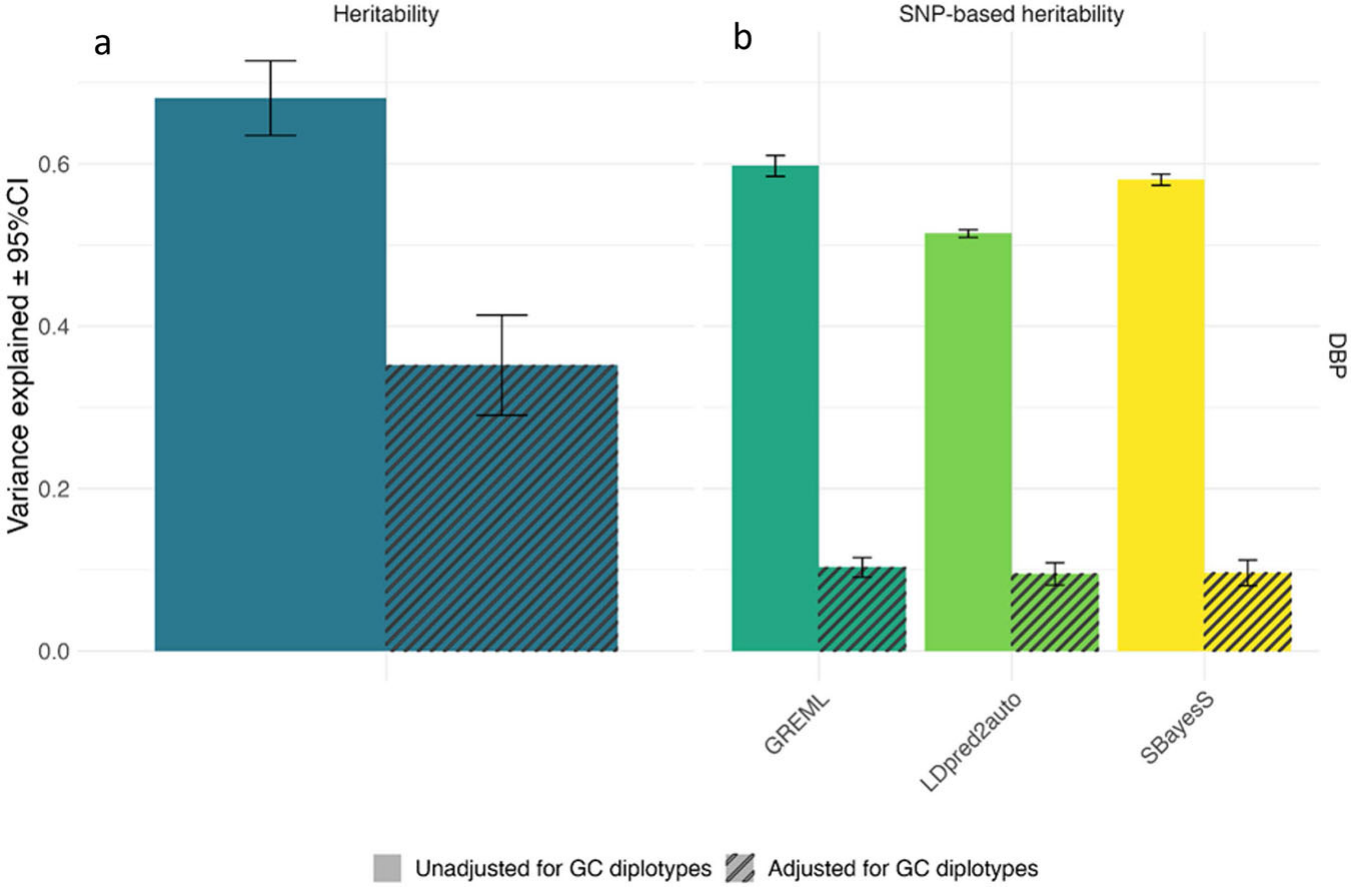
Heritability and SNP-based heritability of DBP. Heritability (**a**) and SNP-based heritability (**b**) estimates for DBP, with and without adjustment for GC haplotypes (adjusted values shown with cross-hatching). The family-based heritability and GREML estimates are based on the European ancestry subset of individuals (*n* = 65,589) and the LDpred2-auto and SBayesS based on the same sample filtered for unrelatedness (*n* = 48,842). Heritability and SNP-based heritability estimates are presented with 95% confidence intervals.

With respect to DBP, the phenotypic variance dramatically declined from 1.0 to 0.47 after the adjustment, because of the substantial contribution of *GC* haplotypes (Fig. 3). Heritability is a ratio statistic, subject to phenotypic variance. Therefore, we reported additional components of heritability. Before the adjustment of GC haplotypes, the heritability (and standard error) of DBP was 0.68 (SE = 0.02), the genetic variance explained by SNPs = 0.58 (SE = 0.01) and the shared environment = 0.10 (SE = 0.02). When adjusted for GC haplotypes, the genetic variance explained by SNPs decreased to 0.05 (SE = 0.002) while the contribution of shared environment was comparable, 0.12 (SE = 0.02) (Supplementary Data 2). These findings indicate that the suggested heritability of DBP is 0.68 and 58% of the variance in DBP was captured by SNPs, 53% attributed to *GC* haplotypes, and 5% attributed to additional genetic variation. These results are consistent with the estimates from SBayesS and LDpred2-auto (Supplementary Data 2) and lend weight to the potential informative value of both the DBP and DBP analysis adjusted for GC haplotypes (henceforth DBP_GC).

### Genome-wide association study (GWAS) analysis and fine mapping

**Fig. 4:**
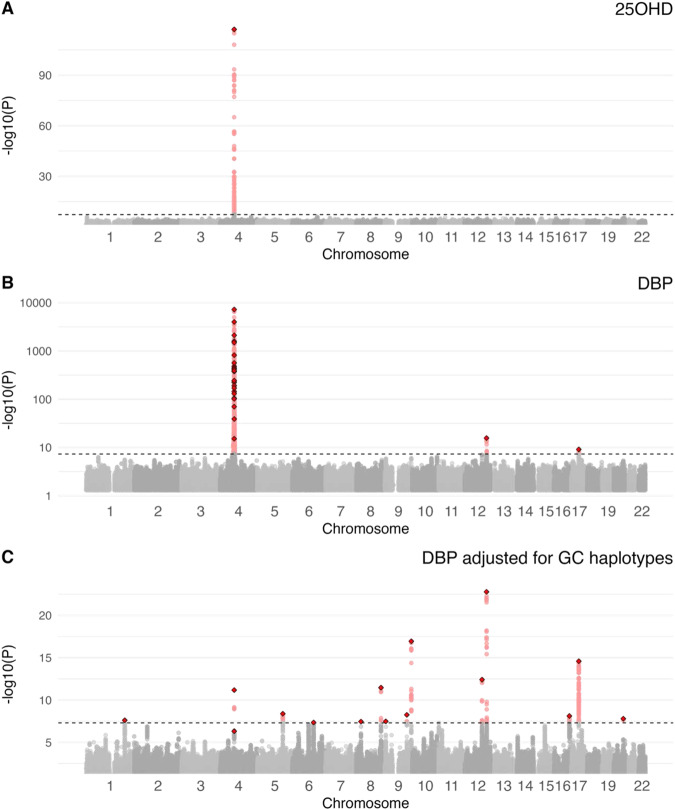
Manhattan plots for 25-hydroxyvitamin D (25OHD), vitamin D binding protein (DBP), and DBP adjusted for GC haplotypes in the iPSYCH case-cohort study. Panel **A** shows the Manhattan plot for 25OHD, with one prominent peak over the GC gene Chromosome 4. Panel **B** shows the Manhattan plot for DBP with a very large peak over the GC gene on Chromosome 4. Panel **C** shows the Manhattan plot for DBP_GC. Note, the Y axis for –log10(*p*) varies between the panels.

A total of 6,091,695 SNPs with MAF ≥0.01 were tested in the GWAS analysis. Based on GCTA–COJO we identified one independent SNP associated with 25OHD concentration, 26 independent SNPs associated with DBP levels (24 of which were on chromosome 4), and 15 independent SNPs (distributed over 10 chromosomes) associated with DBP levels after adjusting for the *GC* haplotypes (Fig. 4). The independent loci for 25OHD located in the *GC* gene on chromosome 4 (rs1352846) had been previously identified^11^. For DBP, we further fine-mapped the genome-wide significant regions in chromosomes 4, 12, and 17 using a combination of PolyFun and SuSiE^42^. For chromosome 4, the key *GC* haplotype-determining rs7041 had a posterior causal probability (PIP) of 1 (Supplementary Data 11). In the GWAS for DBP_GC, an intergenic locus (rs112704913, chromosome 4: 72,571,221 bp, hg19) located 36 kb upstream from the start position of *GC* gene (chromosome 4: 72,607,410-72,669,758 bp, hg19, Ensembl) was also identified (PIP = 0.87). From the 26 COJO-independent hits for DBP, 17 loci were in or close to *GC* (nine upstream, seven within, and one downstream of *GC*, all within a 400 kb range). For chromosome 12, there was a credible set of four SNPs with cumulative PIP >0.95, where the leading SNP rs3184504 (PIP = 0.5) is a missense variant in *SH2B3*. When adjusting DBP for the GC haplotypes, the fine-mapped results decreased the credible set to 3 SNPs, and the PIP of the missense variant increased to 0.78. This shows how the adjusted GWAS increased the power to fine-map potentially causal variants. For chromosome 17 we observed a similar effect. The fine-mapping algorithm did not output a credible set for this region, and the leading SNP rs56030650 (a missense variant in *GSDMA*) had a low PIP of 0.2. Nevertheless, after adjusting for the *GC* haplotypes, the cumulative PIP of the credible set of nine SNPs was 0.95, with the missense variant increasing its PIP to 0.26. FUMA gene-based analysis also showed that the SNPs in *GC*, *SH2B3*, and *GSDMA* were over-represented (Supplementary Data 3). Additional results are shown in Supplementary Data 11). Locus zoom plots for these three regions are shown in Supplementary Figs. 11–13.

### Replication, out-of-sample genetic risk prediction, and sensitivity analysis

We examined if the genetic architecture of 25OHD in our neonatal sample was broadly consistent with that found in the large (*n* = 417,580) UKB adult sample^11^. Of the 143 genome-wide significant COJO SNPs in the UKB 25OHD GWAS, only the most highly significant one was replicated in our 25OHD sample. However, the Pearson’s correlation coefficient between the allele effect sizes for the union of both GWASs genome-wide significant SNPs (i.e., the significant findings from both UKB and the current sample) was 0.66 (*P* value <2.2 × 10^−16^; Supplementary Fig. 11), supporting the hypothesis that the neonatal and adult genetic correlates of 25OHD are broadly comparable.

In order to examine the influence of DBP on 25OHD concentration, we used mtCOJO to condition the UKB 25OHD GWAS summary statistics on our DBP summary statistics. When assessed with and without adjustment for the *GC* haplotypes, we confirmed that the genetic correlates of DBP GWAS were highly influential on 25OHD concentration in an external sample, with only 76 and 79 SNPs out of the 143 COJO SNPs in the UKB 25OHD GWAS remaining genome-wide significant, respectively (Supplementary Data 4).

With respect to out-of-sample prediction (the European sample predicting into the excluded near-European sample), the proportion of DBP variance explained by the effect of the main SNP rs7048 alone from the DBP GWAS was 54%, while adding more SNPs to the polygenic score (PRS COJO, LDpred2-auto, SBayesS) decreased the *r*^2^ to 47%. The maximum variance explained by the P + T PRS was 44% by the smallest threshold *p* value <5e-8, which included 111 SNPs. The proportion of DBP variance explained by the DBP GWAS results adjusted for the GC diplotypes was not significantly different from 0 for any of the PRSs but the SBayesS, which had an *r*^2^ of 9% (Supplementary Data 5).

With respect to the planned sensitivity analyses where we compared GWAS findings based on the entire case-cohort versus the subcohort only, we found that the SNP effect sizes had a Pearson’s correlation coefficient of 0.99 (*P* value <2.2 × 10^−16^, 3839 SNPs) for DBP GWAS and of 0.97 (*P* value <2.2 × 10^−16^, 412 SNPs) for the DBP_GC GWAS. This result supports the hypothesis that the findings based on the overall case-cohort sample are comparable to that found in the nested (smaller) general population sample. GWAS findings from the subcohort are shown in Supplementary Data 6.

### Functional mapping of GWAS findings

We performed gene-based and gene-set analyses, for which results can be found in Supplementary Data 7, 8. As expected, the gene-based analyses identified many genes on chromosome 4 proximal to the *GC* gene. With respect to gene ontology, the top pathway we identified was related to polysaccharide metabolic processes, which may reflect post-translational glycosylation of the DBP protein (a process that influences the properties and elimination of DBP)^4^.

We used SMR to explore the pleiotropic genes that are associated with DBP. Based on both *GC-*adjusted and unadjusted summary statistics, loci on chromosome 17 in close proximity to the adjacent genes *MED24* and *GSDMA* were identified (Supplementary Data 9, 10). These results are consistent with the genes identified by the FUMA gene-based analysis.

### GSMR analyses between DBP and 25OHD

The genetic correlation between summary statistics based on GWAS analyses for unadjusted DBP and 25OHD based on bivariate GREML was 0.58 (SE = 0.05). When estimated at the GWAS summary statistics level with bivariate LDSC regression, it was 0.34 (SE = 0.09) and 0.24 (SE = 0.11) when using the unadjusted and GC-adjusted DBP summary statistics respectively, confirming the substantial contribution of DBP to 25OHD concentration (Supplementary Data 2).

**Fig. 5:**
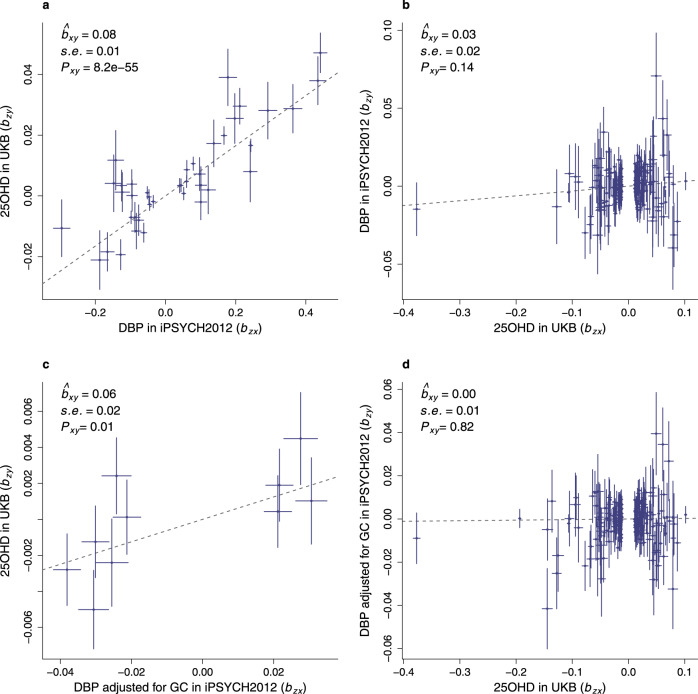
The association of DBP and 25OHD. Panels (**a**, **b**) show the GSMR results of DBP concentration, **a** the effect of DBP on 25OHD (SNPs *n* = 40); and **b** the effect of 25OHD on DBP (SNPs *n* = 201). The GSMR estimates are shown in each panel. The plots show the GSMR estimate of effect, and SE and *P* value, all of which were calculated by GSMR. *P* value was estimated from a two-tailed test. The dashed line represents the GSMR estimate of effect. The summary statistics of DBP is from this study, conducted in iPSYCH2012. The summary statistics of 25OHD is from the study of Revez et al., conducted in UKB. Panels (**c**, **d**) are the GSMR results of DBP concentration adjusted for GC genotypes, **c** the effect of DBP adjusted for GC on 25OHD (SNPs *n* = 10) and **d** the effect of 25OHD on DBP adjusted for GC (SNPs *n* = 222). The GWAS of adjusted DBP was conducted by fitting the GC diplotypes as covariates. The summary statistics of 25OHD are the same as above, from the study of Revez *et al*. All the GSMR analyses were conducted with HEIDI-outlier. The SNPs which were identified as pleiotropy were excluded. The bar shown in the graph represents the GWAS SE at each SNP with its center being the GWAS effect of SNP. The Bonferroni-corrected threshold was 0.025 (=0.05/2).

We used bi-directional GSMR to investigate the relationships between DBP and 25OHD. In the following text, we will focus on the findings with HEIDI filtering (which reduces the potential influence of pleiotropy in the analyses; See Supplementary Data 12 for more details). We found strong support for the hypothesis that high DBP is associated with higher 25OHD levels (Fig. 5). Concerning the forward GSMR (i.e., DBP predicting UKB 25OHD), we found a highly significant association (*b*_*xy*_ = 0.08, SE = 0.005, *P* value = 8.2 × 10^−55^, *N*_SNPs_ = 40). Concerning the reciprocal (reverse) relationship (UKB 25OHD predicting DBP), there was no significant association (*b*_*xy*_ = 0.03, SE = 0.02, *P* value = 0.14, *N*_SNPs_ = 201). After adjusting the *GC* haplotypes, the general pattern of finding persisted (forward GSMR: *b*_*xy*_ = 0.06, SE = 0.02, *P* value = 0.01, *N*_SNPs_ = 10; reverse GSMR: *b*_*xy*_ = 0.003, SE = 0.01, *P* value = 0.82, *N*_SNPs_ = 222). Figure 5b, d suggest that one SNP (rs116970203) may have unduly influenced the findings. The pattern of findings remained unchanged after we repeated these analyses without this SNP (Supplementary Table 3). These results suggest that a 1 SD unit (1.44 ug/L) increase in DBP concentration results in an increase of 0.06–0.08 × SD unit (14.1 nmol/L) of 25OHD concentration (i.e., 0.85–1.13 nmol/L).

### GSMR relationships with other traits

There were no significant associations based on forward or reverse GSMR between GWAS summary statistics based on either DBP or DBP_GC versus any of the neuropsychiatric disorders. With respect to forward GSMR based on summary statistics based on DBP, there were no significant findings for any of the phenotypes.

**Fig. 6:**
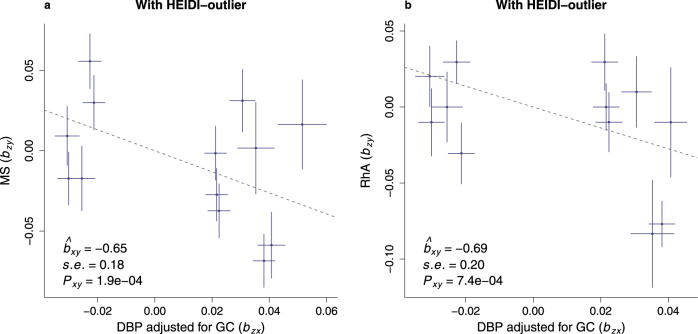
The GSMR effects of DBP concentration on multiple sclerosis (MS) and rheumatoid arthritis (RhA). The GSMR estimates are shown for MS (Panel **a**; SNPs *n* = 13) and RhA (Panel **b**; SNPs *n* = 12). These GSMR estimates include estimate of effect, SE, and *P* value, all of which were calculated by GSMR. The *P* value was estimated from a two-tailed test. The dashed line represents the GSMR estimate of effect. Datasets used in GSMR were from GWAS summary statistics. GWAS of DBP adjusted for GC was conducted in iPSYCH2012. Details of GWAS summary statistics for MS and RhA are provided in Methods. No pleiotropic SNPs were identified by HEIDI-outlier for these two disorders. The bar shown in the graph represents the GWAS standard error for each SNP with its center being the GWAS effect of SNP. The Bonferroni-corrected threshold was 1.9 × 10^−3^ (=0.05/(13 × 2).

With respect to forward GSMR based on GWAS summary statistics for DBP_GC, we found evidence to support causal associations between this phenotype and two autoimmune disorders (Fig. 6a, b). First, we found a negative (i.e., protective) association between DBP_GC and multiple sclerosis (logOR = 0.65, SE = 0.18, *P* value = 1.9 × 10^−4^, *N*_SNPs_ = 13). Second, there was a negative association between DBP_GC and rheumatoid arthritis (logOR = 0.69, SE = 0.20, *P* value = 7.4 × 10^−4^, *N*_SNPs_ = 12). No pleiotropic SNPs were identified by HEIDI-outlier in either multiple sclerosis or rheumatoid arthritis GSMR analyses.

In addition, we found evidence to support the hypothesis that pleiotropic variants may influence the association between DBP_GC and two additional autoimmune disorders (Supplementary Fig. 15a–d). When potentially pleiotropic SNPs were removed, we found a positive association between DBP_GC and a higher risk of Crohn’s disease (logOR = 0.65, SE = 0.19, *P* value = 7.6 × 10^−4^, *N*_SNPs_ = 11). This relationship was not apparent in analyses that included two potentially pleiotropic SNPs (rs11745587, rs56326707; logOR = −0.20, SE = 0.18, *P* value = 0.28, *N*_SNPs_ = 13). In general, the large positive GSMR estimate (when two pleiotropic SNPs were excluded) and the change in sign of the beta estimate when the 2 pleiotropic SNPs were included, suggests that we should be cautious in our interpretation of any potential association between DBP concentration and Crohn’s disease.

**Fig. 7:**
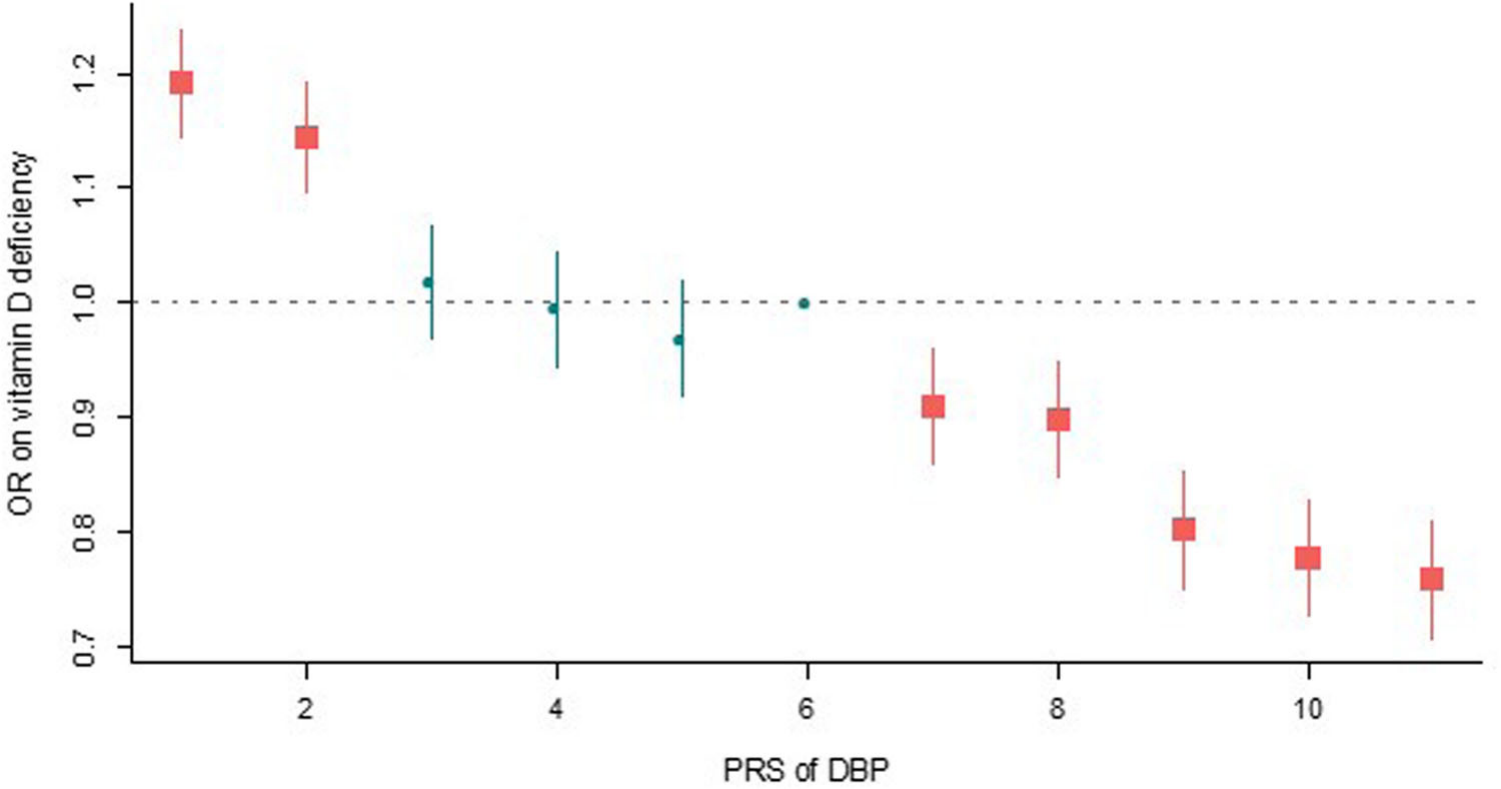
Odds ratio (OR) of DBP PRS on vitamin D deficiency. The PRS of DBP was divided into 11 bins. The sixth bin with the average of PRS is set as the reference category (odds ratio = 1). We conducted a logistic regression for each bin to test the odds ratio of PRS for bin of interest compared to the reference bin. The sample size was 31,615 for each bin. The number of cases/controls of vitamin D deficiency (vitamin D <25 nmol/L) were bin #1 = 4207/24,604, bin #2 = 4058/24,752, bin #3 = 3684/25,105, bin #4 = 3595/25,115, bin #5 = 3525/25,308, bin #6 = 3622/25,251, bin #7 = 3329/25,474, bin #8 = 3307/25,518, bin #9 = 2982/25,820, bin #10 = 2906/26,000, bin #11 = 2823/26,078. The vertical bars represent the 95% confidence interval for the bins (except for the sixth bin which is the reference category). A two-tailed *P* value was estimates for the odds ratio of each bin of interest. *P* values for lower PRS categories (bins 1–5) were 8.9 × 10^−13^, 3.7 × 10^−8^, 0.48, 0.83, and 0.21, respectively. *P* values for higher PRS categories (bins 7–11) were 2.1 × 10^−4^, 3.2 × 10^−5^, 5.1 × 10^−17^, 2.7 × 10^−21^, and 6.3 × 10^−25^, respectively. The Bonferroni corrected threshold was 0.005 (= 0.05/10).

Furthermore, we identified a negative association between DBP_GC and risk of Type 1 diabetes when assessed without the HEIDI-outlier test (logOR = −0.95, SE = 0.17, *P* value = 1.2 × 10^−8^, *N*_SNPs_ = 13 SNPs). When one pleiotropic SNP was removed from the analysis, the association became non-significant (logOR = 0.36, SE = 0.19, *P* value = 0.06, *N*_SNPs_ = 12 SNPs). The identified pleiotropic SNP was rs3184504, a missense variant in *SH2B3*. For both the findings related to Crohn’s disease and Type 1 diabetes, the opposite direction of beta coefficients in the presence or absence of potentially pleiotropic variants weakens the hypothesis that there is a direct influence of DBP_GC on these two disorders. Full details of the GSMR analyses are shown in Supplementary Data 13, 14.

### PheWAS findings

Finally, we examined the association between the two DBP-related summary statistics and phenotypes in the UKB. For the GWAS summary statistics based on DBP, only one finding was significant (after Bonferroni adjustment for multiple comparisons) (Supplementary Data 15). We found a highly significant association with measured 25OHD concentration (part of the UKB biomarker set—Field ID: 30890; beta = 0.09, SE = 0.002, *P* value <1.0 × 10^−100^, *N* = 317,064). Note that the positive effect size (beta value) indicates that variants associated with higher DBP concentrations were associated with higher observed 25OHD concentrations in UKB. Reassuringly, the association with a clinical diagnosis of “vitamin D deficiency” (ICD-10 E55; *N*_cases_ = 3150, *N*_noncases_ = 344,619) was negative and nominally significant (i.e., higher DBP associated with a reduced risk of a clinical diagnosis of vitamin D deficiency; beta = −0.07, standard error = 0.02, *P* value < 1.9 × 10^−4^).

We dichotomized the continuous measure of 25OHD concentration in the UKB according to the Institute of Medicine definition of vitamin D deficiency (i.e., <25 nmol/L)^43^. We then divided the PRS for DBP into 11 bins (quantiles). The sixth (central) bin was set as the reference category. We tested the odds ratio of each binned PRS to the reference bin with logistic regression adjusted for sex, age and 20 PCs. Compared to the reference category, those in each of the five upper quantiles had significantly reduced odds of having vitamin D deficiency, while those in the two lowest quantiles had significantly increased odds of having vitamin D deficiency (Fig. 7 and Supplementary Data 16). Compared to the highest quantile, those in the lowest quantile has 57% increased odds of having vitamin D deficiency (odd ratio = 1.57, 95% confidence intervals 1.49–1.65).

For the DBP_GC DBP summary statistics, we found a range of associated phenotypes (Supplementary Data 17). For example, summary statistics based on DBP_GC were protective for the clinical diagnosis of essential (primary) hypertension (logOR = −0.03, SE = 0.004, *P* value = 2.52 × 10^−11^; *N*_cases_ = 104,892, *N*_non-cases_ = 242,876). Consistent with this finding, DBP_GC was also negatively associated with both observed diastolic and systolic blood pressure in a large UKB subsample (*N* ~253,370, *b*_diastolic_ = −0.02, SE = 0.002, *P* value = 1.7 × 10^−23^; *b*_systolic_ = −0.01, SE = 0.002, *P* value = 5.1 × 10^−11^ respectively). In addition, DBP_GC was associated with: (a) reduced pulse rate (*b* = −0.01, SE = 0.002, *P* value = 8.3 × 10^−9^; *N* = 324,967), (b) gastritis and duodenitis (logOR = −0.03, SE = 0.01, *P* value = 7.5 × 10^−7^; *N*_cases_ = 39,620, *N*_non-cases_ = 308,147); and associated with an increased risk of (c) vasomotor and allergic rhinitis (logOR = 0.03, SE = 0.01, *P* value = 6.08 × 10^−6^; *N*_cases_ = 34,276, *N*_non-cases_ = 313,476), and (d) agranulocytosis (logOR = 0.06, SE = 0.01, *P* value = 1.5 × 10^−5^; *N*_cases_ = 4919, *N*_non-cases_ = 342,850). There was no association between higher DBP_GC and observed 25OHD concentration (*b* = 0.003, SE = 0.002, *P* value = 0.11; *N* = 317,064), but again we found a nominally significant association with a prior clinical diagnosis of vitamin D deficiency (logOR = −0.04, SE = 0.02, *P* value = 0.02; *N*_cases_ = 3150, *N*_non-cases_ = 344,619). Finally, DBP_GC was associated with (a) an education-related measure (higher concentration of DBP associated with more years in education), (b) birthweight (higher concentration of DBP associated with higher birthweight), and (c) two adult anthropometric measures (higher concentration of DBP associated with reduced body fat percentage). PheWAS plots for both the DBP and DBP_GC-based analyses can be found in Supplementary Figs. 16, 17 respectively.

## Discussion

We identified 26 independent loci associated with DBP concentration, 24 of which were either in or in close proximity to the *GC* gene. When we adjusted for key *GC* haplotypes, we identified 15 loci distributed over 10 chromosomes. We confirm the robust influence of *GC*-related variants on the concentration of DBP, and provide clues as to the genetic complexity of this highly polymorphic protein. Mendelian randomization suggests that variants related to increased DBP concentration are associated with higher 25OHD concentration, but not vice versa. Our findings related to autoimmune disorders were of particular interest—Mendelian randomization analyses lend weight to the hypotheses that DBP-related mechanisms influence the risk of multiple sclerosis and rheumatoid arthritis. The following discussion focuses on six key findings.

First, we confirm that the genetic architecture of DBP concentration is characterized by highly influential loci within or near the *GC* gene. In particular, over half (52.6%) of the variance in DBP concentration is explained by two canonical missense variants (rs7041 and rs4588). Consistent with previous literature, we found that the proportions of the different haplotypes varied by genetically-defined ancestry (Supplementary Fig. 10). While each of the six key *GC*-related diplotypes was detected within the group defined as European ancestry, DBP concentrations still showed appreciable variation *within* each of these groups. The genetic correlates underpinning this additional variation were foregrounded in the *GC*-adjusted GWAS, which identified 15 COJO-independent loci distributed over 10 chromosomes (only two were on chromosome 4, in proximity to *GC*).

Second, we show that DBP is highly heritable. Using related individuals, the narrow-sense heritability was 68%. The estimate is similar to the heritability (60%) reported by ref. ^9^. When we examined how much of the variance in DBP concentration could be attributed to common single-nucleotide variants included in the GWAS, the proportions remained appreciable, 53% for the *GC* gene and 5% for the remaining genotypes. The results were consistent across the three methods with different assumptions of genetic architecture, GCTA-GREML, SBayesS, and LDpred2-auto. Moreover, we identified 15 independent COJO loci after the adjustment for the *GC* haplotypes. These 15 COJO SNPs explained a 0.49% variance of DBP in total. The remaining ~4.5% of the variance is likely to be captured by SNPs which were not significant in the current GWAS. These findings suggest DBP has polygenic features, in addition to the very large genetic variance encoded by the *GC* gene. These findings reinforce the value of the *GC*-adjusted GWAS and related post-GWAS analyses.

Third, based on our sample of ~65,000 European-ancestry individuals, we found that the genetic architecture of 25OHD in neonates was consistent with that reported by similar-sized GWAS studies based on adults^10,12^. We found one quasi-independent locus in the *GC* gene. Furthermore, based on the correlation between the effect sizes for the SNPs identified in the UKB-based GWAS (n ~350,000 adults)^11^ and the subset of these SNPs available in our neonatal sample, a significant positive association was found (Pearson *r* = 0.66, *P* value <2.2 × 10^−16^). The family-based heritability for 25OHD was comparable to that reported by Revez et al. in the UKB sample^11^ (current study = 36%; UKB = 32%). It is important to note that neonatal 25OHD concentration is entirely reliant on maternal 25OHD concentrations^44^ and while the correlation between the maternal and offspring genotypes would be 0.5, the genetic correlates of neonatal 25OHD may be more strongly correlated with the (unobserved) maternal genotype, rather than the (observed) neonatal genotype. Because maternal DBP does not cross the placenta^1^, this is not an issue when examining the genetic correlates of DBP in neonates.

Fourth, we identify new candidate loci that influence DBP concentration. As expected with a highly polymorphic protein like DBP, many of the quasi-independent loci (17 of 26 in the main analysis) were in or very close to the *GC* gene (including the canonical missense variant rs7041). Fine-mapping identified: (a) a missense variant in *SH2B3* (rs3184504), which encodes a widely-expressed protein involved in the activation of kinase signaling activities, and which has been linked to a range of disorders, including diabetes^45^, and (b) a missense variant in *GSDMA* (rs56030650) which encodes a precursor of a pore-forming protein that can influence membrane permeabilization. Variants in this gene have been linked to pyroptosis (inflammatory cell death) and inflammatory bowel disease^46^, however, it is currently unclear how variants in this gene may influence DBP concentration.

Fifth, our findings provide convergent evidence that variants that influence DBP concentration influence 25OHD concentration, but not vice versa. Apart from the findings from Mendelian randomization, we found no significant variation in DBP concentration by month of testing (in contrast to 25OHD, which shows marked seasonal variation). The PheWAS results confirm that variants related to higher DBP concentration are associated with (a) a higher concentration of 25OHD and (b) a reduced risk of receiving the clinical diagnosis of vitamin D deficiency. In light of evidence from clinical trials indicating that vitamin D supplementation does not impact DBP concentration^47^, our findings lend additional support to the unidirectional nature of the relationship between DBP and 25OHD.

It has long been appreciated that: (a) variants in the *GC* gene influence the concentration of DBP and (b) that variants within the *GC* gene are robustly and consistently associated with 25OHD concentration. Over the last few decades, there has been a focus on the relationship between (a) total 25OHD (i.e., the value routinely measured by laboratories measures both protein-bound and free 25OHD) and (b) free 25OHD (directly observed by specialized assays or estimated based on prediction models)^2,48^. It is clear that free 25OHD concentration is strongly correlated with the total 25OHD concentration^7^. Our Mendelian randomization findings lend weight to the hypothesis that the higher concentration of DBP is associated with a higher concentration of (total) 25OHD. In light of (a) recent clinical evidence from individuals with homozygous deletions or pathogenic variants of the *GC* gene^5,49^ and (b) findings from *GC* knock-out animal models^6^, the concentration of DBP has been proposed to be a key factor in determining the half-life of vitamin D metabolites, as unbound 25OHD is more rapidly transferred to target cells and catabolised^5,7^. By extension, those with a lower concentration of DBP would be more likely to experience vitamin D deficiency, because this would shorten the functional half-life of 25OHD. A study tracking the excretion of deuterium-labeled 25OHD supports this hypothesis^50^. If two individuals have an identical concentration of total 25OHD (free and bound) at baseline, then in the absence of new vitamin D production, over a given period the individual with a higher concentration of DBP would be less likely to subsequently develop vitamin D deficiency because of the longer half-life of 25OHD. While DBP is not directly involved in pathways leading to the synthesis or catabolism of 25OHD, higher DBP concentration acts as a larger reservoir for 25OHD, extending the effective half-life of 25OHD, and thus provides a more effective ‘buffer’ against future vitamin D deficiency. We speculate that (a) observed lower DBP concentration and/or (b) lower polygene risk scores based on summary scores from DBS-related GWASs, may provide an informative proxy measure related to an increased future risk of vitamin D deficiency. In addition, our findings may cast light on the observation that the concentration of DBP increases substantially during pregnancy^4,51,52^. Increased concentration of DBP may reduce the risk of both maternal vitamin D deficiency and prenatal exposure to developmental vitamin D deficiency.

Sixth, we provide evidence from Mendelian randomization that links DBP concentration and the risk of several autoimmune disorders. There is already a strong body of literature based on observational epidemiology and Mendelian randomization linking low vitamin D status and an increased risk of multiple sclerosis^31,32,53–57^. Based on Mendelian randomization, we also found that increased DBP concentration was associated with a reduced risk of rheumatoid arthritis. In keeping with our findings related to multiple sclerosis, the effect size of this association was substantial (logOR = −0.65, SE = 0.18, *P* value = 1.9 × 10^−4^). There is evidence linking vitamin D deficiency and an increased risk of rheumatoid arthritis^58,59^. The active form of vitamin D (1,25OHD) is an immuno-modulator and has anti-inflammatory effects^60^. Because we identified these autoimmune-related findings only in the summary statistics generated from the DBP_GC analysis (which is a weaker instrument for 25OHD concentration compared to the unadjusted DBP), this raises the possibility that these findings may reflect non-vitamin D-associated properties of DBP (e.g., C5a-mediated chemotaxis, T-cell response, macrophage activation^1,61^). It could also be argued that the established links between 25OHD concentration and the risk of several autoimmune disorders provide a more parsimonious explanation for these particular findings. We hope that our findings can stimulate hypothesis-driven research focused on the role of DBP for multiple sclerosis and rheumatoid arthritis.

Our study has several strengths. Our sample was over 30 times larger than the only other published GWAS of DBP^9^. We were able to assess 25OHD in the same large sample, and also confirm our findings within a subcohort representative of the general population. Our findings also have several important limitations. The sample were neonates at the time of testing, and it remains to be seen if the genetic architecture of DBP identified in our study will generalize to adult populations. We know that certain factors (e.g., pregnancy, use of the oral contraceptive pill) and several disorders that lead to proteinuria can impact DBP concentration in adults^62^. For the assessment of DBP, we used a monoclonal antibody pair, which resulted in a similar bias towards the 1S-isoform of DBP as previously reported in studies comparing Americans of African and Caucasian origin (which also used immunoassays based on monoclonal antibodies)^8,63–66^. However, we restricted the sample to those with European ancestries and conducted the GWAS with and without adjustment for GC haplotypes. Our sample is enriched with people with mental disorders; however, we found no forward or reverse GSMR association between mental disorders and DBP. In addition, we did a planned sensitivity analyses where we ran the GWAS again only in the population-based subcohort (a rare resource that is free of ascertainment bias). The GWAS results in the entire case cohort versus the subcohort sample were comparable. In addition, because our main analyses were restricted to Europeans, there is a need to examine the genetic correlates of DBP concentration in more diverse ancestry groups. Finally, the DBP_GC analysis also identified an SNP (rs635634) that is ~4 kb upstream from the *ABO* gene. We noticed that recent studies of the genetic correlates of the human plasma proteome have identified genes that encode for “master regulator” proteins—variants in these genes have widespread correlations with the concentration of other circulating proteins^67,68^. *SH2B3* and *ABO* were both identified as having associations with over 50 other protein concentrations, thus variants in these genes could directly or indirectly influence generic protein metabolic pathways (e.g. metabolism, protein degradation, and excretion). However, variants in these same genes have also been associated with hematocrit^69^, which may have downstream consequences on the accuracy of a range of sera-based^70^ and dried blood spot-based assays^71^. The influence of genetic variants related to hematocrit on (a) the accurate quantification of blood-based biomarkers, and (b) subsequent GWASs based on these measurements, warrants additional investigation.

Our findings may have clinical consequences—those with genetic variants associated with lower DBP concentrations may have a particular requirement for vitamin D supplementation over the course of winter (compared to those with genetic variants associated with higher DBP concentrations). If our Mendelian randomization findings related to multiple sclerosis and rheumatoid arthritis are replicated in future studies, there may be a case to ensure that those with a genetic predisposition to lower DBP concentrations are encouraged to take regular vitamin D supplements. We hope that the research community will use our findings to examine the relationship between the genetic correlates of DBP concentration and a wider range of disorders. We note with interest that a recent large randomized controlled trial found that vitamin D supplementation reduced the incidence of several autoimmune disorders^33^. If previously completed randomized controlled trials of vitamin D supplementation have access to the genotype of their participants, we speculate the use of supplements may be associated with superior outcomes in those with lower genetically-predicted DBP concentration.

## Methods

### Samples

This study was based on the Lundbeck Foundation Initiative for Integrative Psychiatric Research (iPSYCH) sample^37^, a population-based case-cohort design to study the genetic and environmental factors associated with severe mental disorders. The iPSYCH2012 sample is nested within the entire Danish population born between 1981 and 2005 (*N* = 1,472,762). In total, 86,189 individuals were selected; with 57,377 individuals diagnosed with at least one major mental disorder (schizophrenia, bipolar disorder, depression, autism spectrum disorder (ASD), attention deficit hyperactivity disorder (ADHD)) and a random population cohort of 30,000 individuals sampled from the same birth cohort. By design, there were individuals overlapping between the case sub-cohorts and the random population subcohort. We also included 4791 anorexia nervosa cases (AN; ANGI-DK) from the Anorexia Nervosa Genetics Initiative (ANGI)^72^, which has the same design as iPSYCH2012. Henceforth, we refer to iPSYCH2012 as the combined dataset with the ANGI samples. Blood spots for the individuals included in iPSYCH2012 were obtained from the Danish Neonatal Screening Biobank^73^ and subsequently genotyped and assayed for the concentrations of 25OHD and DBP. Dried blood spot samples have been collected from practically all neonates born in Denmark since 1 May 1981 and stored at −20 °C. Samples are collected 4 to 7 days after birth. Material from these samples has been primarily used for screening for congenital disorders, but are also stored for follow-up diagnostics, screening, quality control, and research. According to Danish legislation, material from The Danish Neonatal Screening Biobank can be used for research after approval from the Biobank, and the relevant Scientific Ethical Committee. There is also a mechanism in place ensuring that one can opt out of having the stored material used for research. Additional details of the Danish Neonatal Screening Biobank are available in the iPSYCH methods paper^37^.

### Blood spot extraction

Two 3.2 mm disks from neonatal dried blood spot (DBS) samples were punched into each well of polymerase chain reaction plates (72.1981.202, Sarstedt). About 130 µL extraction buffer (PBS containing 1% BSA (Sigma Aldrich #A4503), 0.5% Tween-20 (#8.22184.0500, Merck Millipore), and complete protease inhibitor cocktail (#11836145001, Roche Diagnostics)) was added to each well, and the samples were incubated for 1 h at room temperature on a microwell shaker set at 900 rpm. After separating the extract from the filter paper into sterile Matrix 2D tubes (#3232, Thermo Fisher Scientific), the extracts were stored at −80 °C for 6–7 years before analysis. DNA was extracted according to previously published methods^74^. After storage, the protein extracts were aliquoted and were subjected to DBP and 25OHD analysis. Thus, all experimental data originates from a single DBS extraction. Additional details related to blood spot extraction and storage are provided in Supplementary Methods 1.

### Assay of DBP concentration

The extracts were analyzed with a multiplex immunoassay using U-plex plates (Meso-Scale Diagnostics (MSD), Maryland, US) employing antibodies specific for DBP (HYB249-05 and HYB249-01), as well as measuring complement C3 and C4 (results will be reported in a separate manuscript). The antibodies were purchased from SSI Antibodies (Copenhagen, Denmark). Extracts were analyzed diluted 1:70 in diluent 101 (#R51AD, MSD). Capture antibodies (used at 10 ug/mL as input concentration) were biotinylated in-house using EZ-Link Sulfo-NHS-LC-Biotin (#21327, Thermo Fisher Scientific) and detection antibodies were SULFO-tagged (R91AO, MSD), both at a challenging ratio of 20:1. As a calibrator, we used recombinant human DBP #C953 (Bon Opus, Millburn, NJ, USA). Calibrators were diluted in diluent 101 and detection antibodies (used at 1 ug/mL) were diluted in diluent 3 (#R50AP, MSD). Controls were made in-house from part of the calibrator solution in one batch, aliquoted in portions for each plate, and stored at −20 °C until use. The samples were prepared on the plates as recommended by the manufacturer, and were read on the QuickPlex SQ 120 (MSD) 4 min after adding 2x Read buffer T (#R92TC, MSD). Analyte concentrations were calculated from the calibrator curves on each plate using 4PL logistic regression using the MSD Workbench software.

Intra-assay variations were calculated from 38 measurements analyzed on the same plate of a pool of extracts made from 304 samples. Inter-assay variations were calculated from controls analyzed in duplicate on each plate during the sample analysis (1022 plates in total). The lower limit of detection was calculated as 2.5 standard deviations from 40 replicate measurements of the zero calibrator. The higher detection limit was defined as the highest calibrator concentration. The lower and upper detection limits for DBP were 2.07 µg/L and 79.8 mg/L respectively, and the intra-assay and inter-assay coefficient of variance was 7.6 and 22.4% respectively. To validate the stability of the samples during storage, we randomly selected 15–16 samples from five years (1984, 1992, 2000, 2008, and 2016; a total of 76 samples). After extracting the samples and adding them to an MSD plate, the rest of the extracts were frozen for 2 months, thawed and measured as described above to imitate the freeze-thaw cycle of the samples in the study. The oldest samples (from 1984) recorded higher concentrations (Supplementary Fig. 1), most probably due to a change in the type of filter paper after 1989 (Schleicher & Schuell grade 2992 was replaced by Schleicher & Schuell grade 903). In light of this artifact, we adjusted all DBP values by plate (the sequence of testing followed the date of birth of the sample). This is described in further detail below. The protein quantification assays were completed between September 2018 and October 2019. Additional details related to pre-analytic variation are provided in Supplementary Methods 2.

### Assay of 25OHD concentration

Detailed methods for the main assay of 25OHD^75^ and an additional method to correct for exposure to bovine serum albumin^76^ have been published elsewhere. We adapted previously published methods (including comparisons between cord serum and neonatal dried blood spots)^77–80^ in order to assay 25OHD based on protein pellets previously extracted from dried blood spots.

For the assay of 25OHD, 30 µL of each sample was transferred to a Thermo Scientific 96-well polypropylene storage microplates before 120 µL internal standard (reconstituted in acetonitrile and diluted to a working solution of 1:100 compared to the kit insert) was added. After centrifugation, the samples were prepared for a liquid-liquid extraction procedure. About 200 µL of the upper organic phase (containing the purified vitamin D metabolites) was transferred to a Thermo Scientific^TM^ WebSeal Plate+ 96-Well Glass-Coated Microplate. The samples were dried down in an Eppendorf Bench Top Concentrator Plus^TM^ (60 °C) before the vitamin D metabolites were derivatized with 20 µL of the commercial PTAD reagent (reconstituted in ethyl acetate and diluted to a working solution of 1:12). After incubation and quenching (by the addition of 50 µL ethanol), samples were dried down in a concentrator before being reconstituted in 80 µL 1:1 acetonitrile/deionized water solution. After reconstitution, 40 µL was injected into the LC-MS/MS system. The LC system is a Thermo TLX2 Turboflow system, comprised of a CTC Analytics HTS PAL autosampler, a dual LC system (one Agilent 1200 quaternary and one Agilent 1200 binary pump) and two Thermo Scientific hot pocket column heaters. The LC systems are interfaced with a triple quadrupole mass spectrometer (Thermo Scientific TSQ Quantiva) equipped with a heated electrospray ionization probe. The LC system is controlled by Aria MX Direct Control software, whereas the mass spectrometer is controlled by the TSQ Quantiva Tune Application software (version 2.0.1292.15). Thermo TraceFinder^TM^ 3.2 application software is used to acquire and process data.

The development of the new assay was validated following the Clinical and Laboratory Standards Institute´s approved guideline for liquid chromatography-mass spectrometry methods (C62-A) ((CLSI), 2014). Intermediate precision was obtained by quantifying the concentration of three stable isotope labeled external quality controls (PerkinElmer) with a low, medium and high concentration of each vitamin D metabolite. To examine intra- and inter-assay precision we used control samples from adult volunteers and examined triplicate samples within one assay run, and also examined these samples on three consecutive days, respectively. In keeping with best practice, we used Standard Reference Material (Vitamin D Metabolites in Frozen Human Serum - SRM® 972 - from NIST). This material was mixed with purified erythrocytes and then transferred onto filter paper. Based on these samples, the accuracy of the assay was between 92 to 105% and the coefficient of variance ranged from 4.7 to 13.2%. The relative errors ranged from −7.9 to 5.7%. In order to determine the lowest level of quantification, dilutions of the lowest stable isotope-labeled calibrator standards for both vitamin D metabolites (2H6-25OHD2 and 2H6-25OHD3) were prepared and quantified. The method was able to reliably detect a concentration of both 25OHD2 and 25OHD3 down to approximately 5 nmol/L in full blood. All analyses were based on a total of 25OHD (the sum of 25OHD2 and 25OHD3). In addition, our laboratory participates in the Vitamin D External Quality Assessment Scheme (DEQAS)^81^. During the period when the iPSYCH samples were analyzed (November 2018 to February 2021), our laboratory assessed 9 panels of 5 DEQAS standard reference samples (total samples *n* = 45). Based on these samples, the mean (and range) bias from the target values was 3.8% (−10.6, 12.6).

### Genotyping and quality control

Individuals included in iPSYCH2012 were genotyped using the Infinium PsychChip v1.0 array (Illumina, San Diego, CA, USA). In total, 80,873 individuals were successfully genotyped across 26 waves for ~550,000 variants^37^. We excluded SNPs with minor allele frequency (MAF) <0.01, Hardy–Weinberg equilibrium (HWE) *p* value <1 × 10^−5^ or non-SNP alleles (i.e., insertions and deletions, INDELs). About 245,328 autosomal SNPs were retained in the backbone set. The backbone set was used to impute the genotypes with the Haplotype Reference Consortium reference panel^82^ following the RICOPILI pipeline^83^. Imputed best guess genotypes were further filtered for imputation quality (INFO score >0.8), genotype call probability (*P* > 0.8), missing variant call rates <0.05, Hardy–Weinberg equilibrium (HWE) *P* value ≥1 × 10^−5^ and minor allele frequency (MAF) >0.01, resulting in 6,091,695 variants remaining.

Darker skin color can reduce actinic production of vitamin D, and because non-European ancestry is associated with variants in DBP (which can influence protein concentration), our primary analyses were in those with European ancestry. We performed principal component analysis (PCA) following ref. ^84^. The genetic ancestry of the samples was inferred using R packages bigsnpr and bigutilse following ref. ^85^, where 73,645 individuals were classified as having European ancestry. The genetic relationship matrix (GRM) of the individuals was estimated by GCTA v1.93^86^. There were 57,747 unrelated individuals with a pairwise coefficient of genetic relationships <0.05.

### Phenotype distributions and covariates

From the 77,482 individuals with genetic data, 71,944 and 71,212 had DBP and 25OHD measurements respectively. The DBP and 25OHD metabolites were quantified in 1030 and 1010 plates, respectively. The quantification plates for DBP and 25OHD explained 11.8 and 55.6% of the phenotypic variance respectively. Note that the sequence of testing followed the date of birth, so the marked seasonal variation in 25OHD concentration would be captured in the between-plate variance. We used linear mixed models to pre-regress the effect of the quantification plates from DBP and 25OHD and applied a rank-based inverse-normal transformation (RINT) to the model residuals. The raw distributions of the neonatal DBP and 25OHD can be seen in Supplementary Fig. 6. For DBP in the entire sample, the mean (and standard deviation) was 2.24 (1.44) µg/L (median and interquartile range: 2.00, 1.19–2.98 µg/L). For DBP in the European subsample, the mean (and standard deviation) was 2.25 (1.44) µg/L (median and interquartile range: 2.01, 1.21–2.99 µg/L). We examined the association between (a) sex, year and month of birth, gestational age, maternal age, and (based on infant genotype) the first 20 principal components (PCs) on (b) 25OHD and DBP concentrations. After, adjusting for the plate effect, none of these variables were significantly associated with DBP levels, while the month of birth, year of birth, gestational age, and maternal age were still significantly associated with 25OHD levels. Additional details for all covariate associations and distributions can be found in Supplementary Data 1.

### Genome-wide association study (GWAS) analyses

To identify genetic variants associated with neonatal DBP and 25OHD blood concentrations, we performed a linear mixed model GWAS implemented in fastGWA^87^ on the subset of European ancestry individuals (*N*_DBP_ = 65,589, *N*_25OHD_ = 64,988). After pre-adjusting for the quantification plates, we fitted sex, year of birth, genotyping wave and the first 20 PCs as covariates in the model in the DBP genetic analyses, and additionally month of birth, gestational age and maternal age in the 25OHD genetic analyses. In light of the strong influence of the *GC* haplotypes of DBP concentration^9^, and the potential haplotype-related bias in our monoclonal assay^8^, we also performed a GWAS adjusted for the 6 *GC* diplotypes, which were fitted as a covariate in the fastGWA model. Henceforth, we will label the two DBP GWASs and related post-GWAS analyses as (a) DBP (unadjusted GWAS) and (b) DBP_GC (GWAS for DBP adjusted for *GC* haplotypes).

To identify independent associations, we conducted a conditional and joint (COJO; GCTA–cojo-slct) analysis^88^ using default settings and the European ancestry subset of individuals as LD reference. In addition, we conducted a multi-trait conditional and joint (mtCOJO) analysis^89^ to condition results from the UK Biobank (UKB) 25OHD GWAS^11^ on (a) DBP and (b) DBP_GC with fastGWA.

The iPSYCH case-cohort study is enriched with individuals with psychiatric disorders (i.e., the cases) but also contains a uniform randomly-selected population-based subcohort. To explore if case-enrichment in the sample may have biased the findings from the GWAS, as a planned sensitivity analysis, we ran the GWAS again only within the population-based subcohort. Based on the union of the genome-wide significant loci from the entire case-cohort and the subcohort samples, we examined the correlation between the effect sizes (beta values) using Pearson’s correlation coefficients^90^.

### Heritability and SNP-based heritability

Our sample had 23,126 individuals that shared at least one off-diagonal GRM value >0.05, of which 6313 had a (off-diagonal) GRM value >0.2 with at least one other individual in the sample. We estimated the heritability of both 25OHD and DBP using methods described by ref. ^41^, within the subset with European ancestry. This method estimates pedigree-based and SNP-based heritability simultaneously in one model using family data and is implemented in GCTA^86^.

Finally, we estimated the SNP-based heritability using LD-score regression^91^, SBayesS^92^, and LDpred2-auto^93^ from the GWAS summary statistics. We also estimated the polygenicity (p) parameter with SBayesS and LDpred2-auto. In order to derive these estimates, we used linear regression GWAS summary statistics from unrelated European individuals (*N*_DBP_ = 48,842, *N*_25OHD_ = 48,643) and filtered down to the intersection with the HapMap3 set of variants (https://www.sanger.ac.uk/resources/downloads/human/hapmap3.html).

### Fine-mapping and functional annotation

Fine-mapping of the GWAS summary statistic results was performed using a combination of (a) PolyFun^42^ for computing prior causal probabilities based on functional annotations and (b) SuSiE^94^ which fine-maps the variants and provides posterior inclusion probabilities (PIPs) and credible sets of variants. First, we estimated truncated per-SNP heritabilities for both our GWAS summary statistics (DBP and DBP_GC) using the L2-regularized S-LDSC method described in PolyFun for the set of coding, conserved, regulatory and LD-related annotations described in ref. ^95^ The LD-scores for these annotations were computed using our subset of European ancestry individuals belonging to the subcohort (*N* = 24,324). We then used the truncated per-SNP heritabilities as prior causal probabilities in SuSiE for fine-mapping. We only performed fine-mapping on the genome-wide significant loci on the DBP GWAS summary statistics. The credible sets obtained in SuSiE were functionally annotated using the Ensembl Variant Effect Predictor (VEP) v85^96^.

### Genetic ancestry inference

By design, the iPSYCH case-cohort samples are born in Denmark. To infer their genetic ancestry we used the sample’s parental country of birth as a proxy, as determined by the Danish Registers. First, we identified the subset of individuals in which both parents were born in the same region (“Africa”, “Asia”, “Australia”, “Denmark”, “Europe”, “Greenland”, “The Middle East”, “N.America”, “S.America”, and “Scandinavia”). The regions “Denmark”, “Europe”, “N.America”, “S.America”, “Scandinavia”, and “Australia” were all re-defined as “Europe”. We then looked at the country of birth of the father and kept only countries where there were >10 individuals born in that country.

Using the father’s country of birth as the grouping variable, we calculated the geometric median of the first 20 principle components (PCs) per country. Then we calculated the distance to all country centers and applied a hierarchical clustering algorithm (base r hclust function with method = “single”). The population centers were then chosen based on a visual inspection of the clusters as the country with the largest sample size. The following countries were chosen as population centers: “Turkey”, “Kingdom of Morocco”, “Islamic Republic of Pakistan”, “Denmark”, “The Somali Republic”, “The Socialist Republic of Vietnam”, and “The Gambia”. After choosing the cluster centers, all other samples were assigned to the nearest cluster inside a threshold defined as thr_sq_dist = 0.002 × (max(dist(all_centers)^2)/0.40) (Supplementary Fig. 2). The cluster tags were changed from country names to geographical region names, as individuals from nearby countries where clustered together in the final classification. The PC1 vs. PC2 plot of the different ancestry clusters is shown in Supplementary Fig. 3.

### Out-of-sample genetic risk prediction

From the European ancestry definition described above, we identified a replication sample of nearly-European individuals by expanding the threshold around the center of the European cluster to thr_sq_dist = 0.002 × (max(dist(all_centers)^2)/0.10) (Supplementary Fig. 4). This resulted in a sample of 1881 individuals of nearly-European ancestry. From these, we identified 1529 individuals not related to each other or to anyone in the main analysis (i.e., all GRM off-diagonals <|0.05|). Supplementary Fig. 5 shows the PC1 vs. PC2 plot of the replication sample compared to the other ancestry clusters.

These individuals were used as a pseudo-replication sample to examine the out-of-sample prediction accuracy of polygenic risk scores (PRSs). The PRS for 25OHD was computed with SBayesR^97^ and downloaded from the PGS Catalog (ID PGS000882)^98^. The PRSs for the four phenotypes (DBP, 25OHD and these two adjusted for the *GC* haplotypes) were constructed using SBayesS^92^ and LDpred2-auto^93^ from our set of GWAS summary statistics. We used linear regression GWAS summary statistics (with the sample filtered for relatedness) for the PRS methods. For SBayesS, we used the provided UKB HapMap3 shrunk sparse LD matrix as an LD reference. For LDpred2-auto, we used the LD blocks based on the subset of HapMap3 variants provided in the paper as LD reference.

We also calculated PRSs using the independent SNP weights estimated by COJO^88^ and the clumping threshold (C + T) method with window size 250 kb and *r*^2^ < 0.1 (M = 201,402 SNPs)) and *P* value thresholds (5 × 10^−8^, 1 × 10^−6^, 1 × 10^−4^, 0.001, 0.02, 0.05, 0.1, 0.2, 0.5, 1). The prediction models examined the phenotypic variance explained (r2) after adjusting for sex, age, and the first 20 PCs.

### Genetic correlations

The genetic correlation between 25OHD and DBP was estimated in a bivariate GREML analysis (GCTA–reml-bivar) and from GWAS summary statistics with bivariate LD-score regression^99^.

### FUMA, GSMR, SMR, and PheWas

Functional mapping and annotation of genome-wide association studies (FUMA)^100^ was used to examine gene-based and gene-set analyses. We conducted generalized summary-based Mendelian randomization (GSMR)^89^ to explore the causal relationship between (a) DBP and 25OHD blood concentrations and (b) between DBP concentration and a range of psychiatric and cognitive phenotypes (schizophrenia, major depression, bipolar disorder, ASD, ADHD, Alzheimer’s disease, and educational attainment), and with selected autoimmune disorders (multiple sclerosis, amyotrophic lateral sclerosis, type 1 diabetes, Crohn’s disease, ulcerative colitis, and rheumatoid arthritis). All the relevant GWAS summary statistics are publicly available (schizophrenia^101^, major depression^102^, bipolar disorder^103^, autism spectrum disorder^104^, attention deficit hyperactivity disorder^105^, Alzheimer’s disease^106^, educational attainment^107^, multiple sclerosis^108^, amyotrophic lateral sclerosis^109^, type 1 diabetes^110^, Crohn’s disease^111^, ulcerative colitis^111^, and rheumatoid arthritis^112^). As the effect of DBP and 25OHD on these phenotypes may be driven by pleiotropy, the analyses were conducted with and without applying the heterogeneity in dependent instrument (HEIDI) outlier method, which removes loci with strong putative pleiotropic effects^89^. We randomly sampled 10,000 unrelated European individuals from iPSYCH2012 as the LD reference cohort. We used a Bonferroni-corrected threshold of 1.9 × 10^−3^ (0.05/(13 × 2)) in the GSMR analysis.

We performed summary-data-based MR (SMR) to identify genes with causal/pleiotropic effects on DBP, using the eQTL data from GTEx v8^113^. For this analysis, we used the same LD reference cohort as used in the GSMR analysis. In total, there were 195,904 probes from 49 tissues. We accounted for multiple testing by using a Bonferroni-corrected threshold of 2.6 × 10^−7^ (0.05/195,904).

The PheWAS analysis was conducted in the UKB using; (1) linear model, *y*_*j*_ = *x*_*j*_ + *c*_*j*_ + *e*_*j*_ for quantitative traits or (2) logistic model, logit(*y*_*j*_) = *x*_*j*_ + *c*_*j*_ + *e*_*j*_ for dichotomous traits, where *y*_*j*_ represents phenotype in UKB, *x*_*j*_ represents the polygenic score of DBP or DBP adjusted for *GC* genotypes, and *c*_*j*_ represents the covariates. There were 1149 phenotypes included in the PheWAS analysis, 1027 diseases, 52 anthropometric and brain imaging measures, and 70 infectious disease antigens. The diseases were classified by using the International Classification of Diseases, 10th version (ICD-10) code. The quantitative traits were normalized using RINT with mean 0 and variance 1. The PRSs were generated using SBayesR^97^ with the reference LD matrix estimated from 1,145,953 HapMap3 SNPs in the UKB. PRSs were computed for 348,501 individuals of European ancestry. The individuals were genetically unrelated (relationship <0.05). The covariates included in the model were sex, age and 20 PCs. The significance threshold used was 4.4 × 10^−5^ (0.05/1149).

### Ethics and data approvals

The study was approved by the Danish Data Protection Agency, and data access was approved by Statistics Denmark and the Danish Health Data Authority. Approval by the Ethics Committee and written informed consent were not required for register-based projects [Act no. 1338 of 1 September 2020, section 10 on research ethics for administration of health scientific research projects and health data scientific research projects]. All data were de-identified and not recognizable at an individual level.

### Reporting summary

Further information on research design is available in the Nature Portfolio Reporting Summary linked to this article.

### Supplementary information

Supplementary Information

Description of Additional Supplementary Files

Supplementary Data 1-17

Reporting Summary

## Data Availability

The data generated during this study are included in this published article and its supplementary files, with the exception of the person-level data and code related to the iPSYCH sample (including DBP and 25OHD concentrations, genotypes, clinical, and demographic data). Owing to the sensitive nature of these iPSYCH data (which includes the ANGI subsample), individual-level data can be accessed only through secure servers where the download of individual-level information is prohibited. Each scientific project must be approved before initiation, and approval is granted to a specific Danish research institution. International researchers may gain data access through collaboration with a Danish research institution. More information about getting access to the iPSYCH data can be obtained at https://ipsych.dk/en/about-ipsych. eQTL data were based on GTEX version 8 (the data used for the analyses described in this manuscript were obtained from the GTEx Portal on 02/01/22). Summary statistics from the following studies were used in the GSMR analyses and are publicly available: schizophrenia^101^, major depression^102^, bipolar disorder^103^, autism spectrum disorder^104^, attention deficit hyperactivity disorder^105^, Alzheimer’s disease^106^, educational attainment^107^, multiple sclerosis^108^, amyotrophic lateral sclerosis^109^, type 1 diabetes^110^, Crohn’s disease^111^, ulcerative colitis^111^, and rheumatoid arthritis^112^. We used data from UK Biobank https://www.ukbiobank.ac.uk/ Application Number 12505. The summary statistics from the GWAS for 25OHD, DBP, and DBP adjusted for GC haplotypes are available via the GWAS Catalog https://www.ebi.ac.uk/gwas/ (Accession numbers GCST90162562, GCST90162563, and GCST90162564).
