# Publisher Correction: Genetic correlates of vitamin D-binding protein and 25-hydroxyvitamin D in neonatal dried blood spots

**DOI:** 10.1038/s41467-024-46199-7

**Published:** 2024-02-26

**Authors:** Clara Albiñana, Zhihong Zhu, Nis Borbye-Lorenzen, Sanne Grundvad Boelt, Arieh S. Cohen, Kristin Skogstrand, Naomi R. Wray, Joana A. Revez, Florian Privé, Liselotte V. Petersen, Cynthia M. Bulik, Oleguer Plana-Ripoll, Katherine L. Musliner, Esben Agerbo, Anders D. Børglum, David M. Hougaard, Merete Nordentoft, Thomas Werge, Preben Bo Mortensen, Bjarni J. Vilhjálmsson, John J. McGrath

**Affiliations:** 1 National Centre for Register-Based Research, Aarhus University, 8210 Aarhus V, Denmark; 2 Center for Neonatal Screening, Department of Congenital Disorders, Statens Serum Institut, Copenhagen, Denmark; 3 Clinical Mass Spectrometry, Statens Serum Institut, Artillerivej 5, DK-2300 Copenhagen S, Denmark; 4 Testcenter Denmark, Statens Serum Institut, Artillerivej 5, DK-2300 Copenhagen S, Denmark; 5 Institute for Molecular Bioscience, University of Queensland, Brisbane, QLD Australia; 6 Queensland Brain Institute, University of Queensland, Brisbane, QLD Australia; 7 The Lundbeck Foundation Initiative for Integrative Psychiatric Research, iPSYCH, 8210 Aarhus V, Denmark; 8 Department of Psychiatry, University of North Carolina at Chapel Hill, Chapel Hill, NC USA; 9 Department of Medical Epidemiology and Biostatistics, Karolinska Institutet, Stockholm, Sweden; 10 Department of Nutrition, University of North Carolina at Chapel Hill, Chapel Hill, NC USA; 11 Department of Clinical Epidemiology, Aarhus University and Aarhus University Hospital, 8200 Aarhus N, Denmark; 12 Department of Affective Disorders, Aarhus University and Aarhus University Hospital-Psychiatry, Aarhus, Denmark; 13 CIRRAU - Centre for Integrated Register-based Research, Aarhus University, Aarhus, Denmark; 14 Center for Genomics and Personalized Medicine, Aarhus University, Aarhus, Denmark; 15 Department of Biomedicine and the iSEQ Center, Aarhus University, Aarhus, Denmark; 16 Department for Congenital Disorders, Statens Serum Institut, 2300 Copenhagen S, Denmark; 17 Mental Health Services in the Capital Region of Denmark, Mental Health Center Copenhagen, University of Copenhagen, 2100 Copenhagen, Denmark; 18 Department of Clinical Medicine, University of Copenhagen, 2200 Copenhagen N, Denmark; 19 Institute of Biological Psychiatry, Mental Health Services, Copenhagen University Hospital, Copenhagen N, Denmark; 20 Department of Clinical Medicine, University of Copenhagen, Copenhagen, Denmark; 21 Lundbeck Center for Geogenetics, GLOBE Institute, University of Copenhagen, Copenhagen, Denmark; 22 Bioinformatics Research Centre, Aarhus University, 8000 Aarhus C, Denmark; 23 Queensland Centre for Mental Health Research, The Park Centre for Mental Health, Brisbane, QLD 4076 Australia

**Keywords:** Genetics research, Epidemiology

Correction to: *Nature Communications* 10.1038/s41467-023-36392-5, published online 15 February 2023

The original version of this article contained an error in the second paragraph of the ‘Assay of DBP concentration’ section of the ‘Methods’, which incorrectly read ‘The lower and upper detection limits for DBP were 2.07 and 79.8 mg/L respectively’. The correct version states ‘2.07 µg/L’ in place of ‘2.07’. This has been corrected in both the PDF and HTML versions of the article.

