# Supplementary material for: Genetic correlates of vitamin D-binding protein and 25-hydroxyvitamin D in neonatal dried blood spots
Source: Nat Commun. 2023 Feb 15;14:852. doi: 10.1038/s41467-023-36392-5 (PMC9932173; doi:10.1038/s41467-023-36392-5)
Supplement: Supplementary file 1 — Supplementary Information [file 41467_2023_36392_MOESM1_ESM.pdf]

SUPPLEMENTARY MATERIAL

Genetic correlates of vitamin D-binding protein and 25-hydroxyvitamin D in neonatal dried blood spots

Contents

SUPPLEMENTARY TABLES 3

Supplementary Table 1 GC haplotypes........ 3

Supplementary Table 2 GC-based haplotype combinations (diplotypes). 3

Supplementary Table 3 Reverse GSMR estimates of 25OHD on DBP, with and without rs116970203 4

SUPPLEMENTARY FIGURES 5

Supplementary Figure 1 Boxplots showing the median value of DBP concentration split by year of birth 5

Supplementary Figure 2 Genetic ancestry - Histogram of distance to cluster centre........ 6

Supplementary Figure 3 Genetic ancestry – plot of principal component 1 (PC1) versus principal component 2 (PC2) 7

Supplementary Figure 4 Defining the near-European replication sample – distance to centre cluster 8

Supplementary Figure 5 Defining the near-European replication sample – principal component 1 (PC1) versus principal component 2 (PC2)........ 9

Supplementary Figure 6 Distribution of 25OHD and DBP before and after transformation 10

Supplementary Figure 7 Correlation between vitamin D binding protein (DBP and 25-hydroxyvitamin D (25OHD)........ 11

Supplementary Figure 8 Seasonal variation – 25-hydroxyvitamin D (25OHD and vitamin D binding protein (DBP) concentration by month of birth 12

Supplementary Figure 9 DBP and 25OHD concentration by GC haplotypes combinations (diplotypes) in the full sample (European and non-European ancestry)........ 13

Supplementary Figure 10 GC diplotypes by inferred ancestry groups........ 14

Supplementary Figure 11 Fine mapping of candidate regions – chromosome 4........ 15

Supplementary Figure 12 Fine-mapping of candidate region – chromosome 12 16

Supplementary Figure 13 Fine-mapping of candidate region – chromosome 17 17

Supplementary Figure 14 Correlation between the SNP estimates for the 25-hydroxyvitamin D (25OHD) GWAS estimates based on the iPSYCH case-cohort versus UK Biobank samples........ 18

Supplementary Figure 15 Mendelian randomization between GWAS summary statistics for DBP condition on GC (DBP_GC) versus Crohn's disease (CD) and type 1 diabetes (T1D) 19

Supplementary Figure 16 Phenome-wide association study (PheWAS) analysis of vitamin D binding protein (DBP) summary statistics in the UK Biobank........ 20

Supplementary Figure 17 Phenome-wide association study (PheWAS) analysis of vitamin D binding protein (DBP) adjusted for GC haplotypes summary statistics in the UK Biobank 21

SUPPLEMENTARY TABLES

Supplementary Table 1 GC haplotypes

The individual's GC gene haplotypes were constructed based on the combination of the GC isoforms (not phased data). The GC haplotypes can be inferred from the specific allele combination of the two SNPs rs4588 and rs7041 as GC 1f (rs7041-T & rs4588-C), GC 1s (rs7041-G & rs4588-C) and GC 2 (rs7041-T & rs4588-A). Both the SNPs were hard-called genotyped for the iPSYCH data, as they are included in the PsychChip v1.0 array. Table S1 shows the coding of the SNPs in the chip.

CHR	SNP	POS (GRCh37)	freq	A1	A2
4	rs4588	72618323	0.727	G	T
4	rs7041	72618334	0.428	A	C

Supplementary Table 2 GC-based haplotype combinations (diplotypes).

Both SNPs have been coded using the opposite strand to the isoform definition, but we defined the haplotypes based on the genotype call in iPSYCH. The code indicates the number of copies of the alternative allele (A1). For the double heterozygous combination or the SNPS rs7041 and rs4588, where rs7041-CA and rrs48-TG there are 3 different resulting haplotypes CT/AG, CG/AT and AT/CG (note two combinations are indistinguishable without phased data). The AG haplotype is rare, thus the three common haplotype variants (CT, CG, and AT) formed six diplotypes.

Haplo type	Code rs7041	Genotype rs7041	Code rs4588	Genotype rs4588
1S/1S	0	CC	2	GG
1S/2	1	CA	1	TG
1F/1S	1	CA	2	GG
2/2	2	AA	0	TT
1F/2	2	AA	1	TG
1F/1F	2	AA	2	GG

Supplementary Table 3 Reverse GSMR estimates of 25OHD on DBP, with and without rs116970203

The HEIDI-outlier was applied in the test. Raw – all clumped SNPs were included. SNP-excluded - rs116970203 was excluded in the GSMR analysis. Mendelian randomisation analysis estimates from GWAS results from 25OHD and DBP, obtained with generalized summary mendelian randomization (GSMR)[1] . The GWAS on 25OHD UKB refers to Revez et al [2]. bxy, GSMR estimated effect size of the exposure on the outcome; se, standard error of bxy; p, P-value of bxy (two-sided P value); the Bonferroni corrected threshold was 1.25×10^{-2} (= 0.05/4). nsnp, number of genetic instruments used in the GSMR analysis.

Exposure	Outcome	Scenario	$\hat{b}_{xy}$	*SE*	*P*-value	N_{SNP}
25OHD	DBP	Raw	0.031	0.021	0.14	201
25OHD	DBP	SNP-excluded	0.040	0.023	0.08	200
25OHD	DBP adjusted for GC	Raw	0.003	0.012	0.82	222
25OHD	DBP adjusted for GC	SNP-excluded	0.006	0.013	0.65	221

SUPPLEMENTARY FIGURES

Supplementary Figure 1 Boxplots showing the median value of DBP concentration split by year of birth

Boxplots showing the median value of DBP concentration split by year of birth (x-axis). Y-axis displays concentration in mg/L. The centre lines show the medians; box limits indicate the 25th and 75th percentiles; whiskers extend 1.5 times the interquartile range from the 25th and 75th percentiles. The individual samples (including the outliers) are represented by dots.

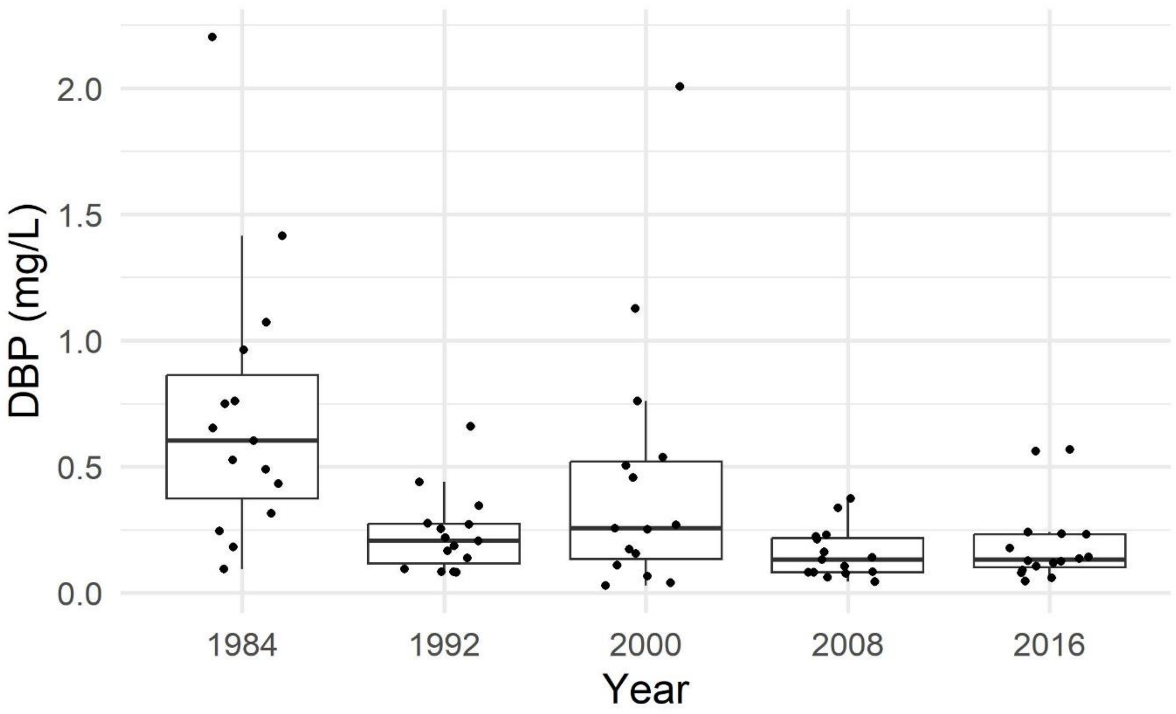

Supplementary Figure 2 Genetic ancestry - Histogram of distance to cluster centre

Histogram showing the frequency (y axis) of genetic ancestry subgroups defined by distance from the ancestry-derived cluster centre (x axis). Subjects below the red vertical reference line were defined as European ancestry.

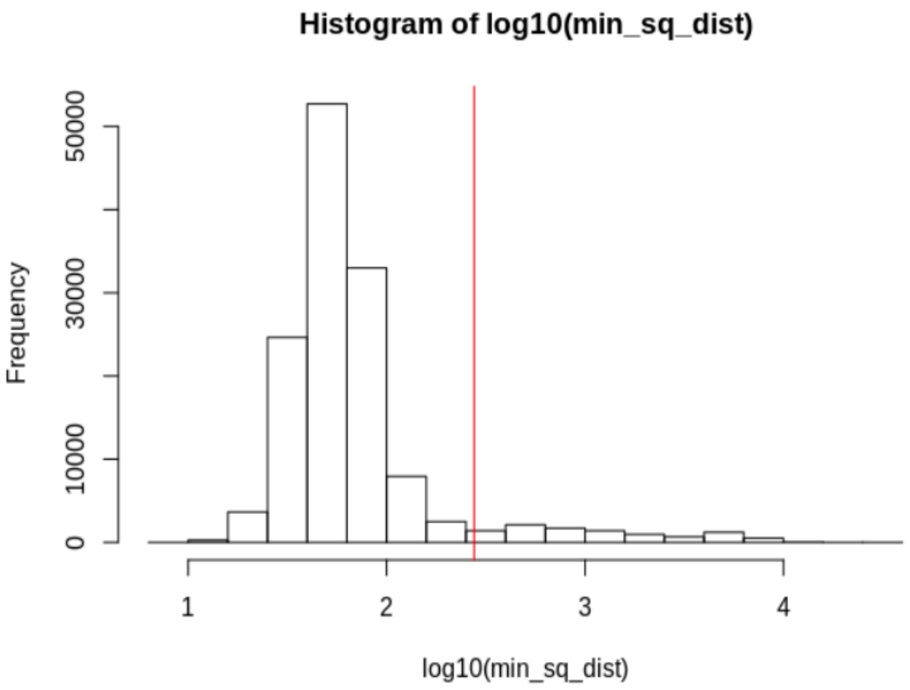

Supplementary Figure 3 Genetic ancestry – plot of principal component 1 (PC1) versus principal component 2 (PC2)

Scatterplot showing the distribution of subjects according to Principal Component 1 (PC1) versus Principal Component 2 (PC2), but putative geographical region of ancestry origin.

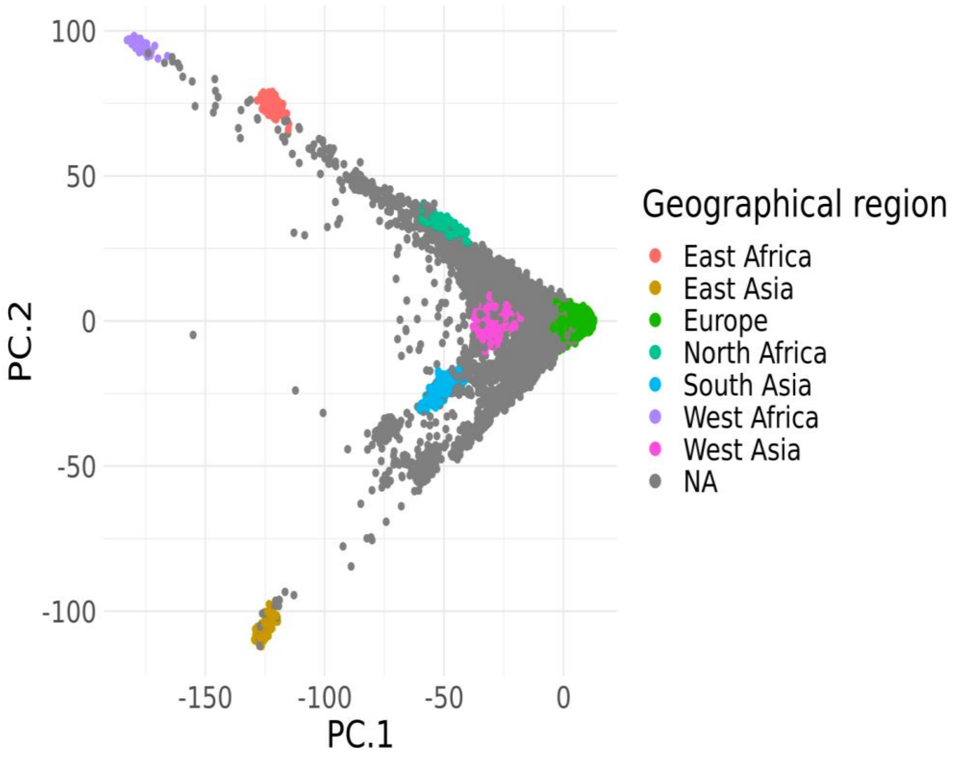

Supplementary Figure 4 Defining the near-European replication sample – distance to centre cluster

Histogram showing the frequency (y axis) of genetic ancestry subgroups defined by distance from the ancestry-derived cluster centre (x axis). The subjects between the red and blue vertical reference were defined as near-European for the independent replication sample.

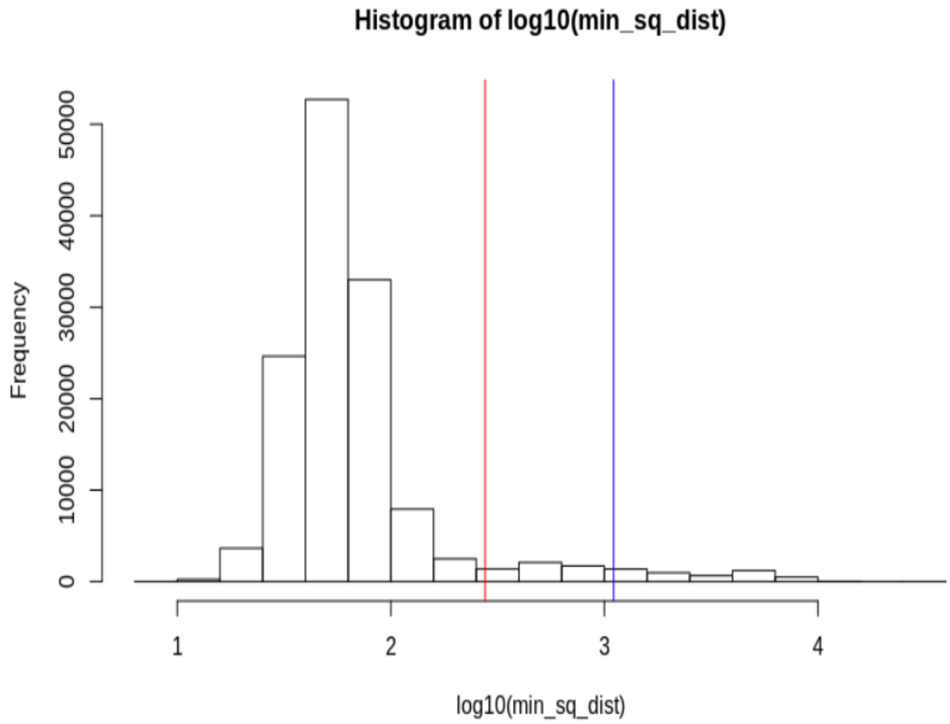

Supplementary Figure 5 Defining the near-European replication sample – principal component 1 (PC1) versus principal component 2 (PC2)

Scatterplot showing the distribution of subjects within the near-European replication sample, according to Principal Component 1 (PC1) versus Principal Component 2 (PC2), but putative geographical region of ancestry origin.

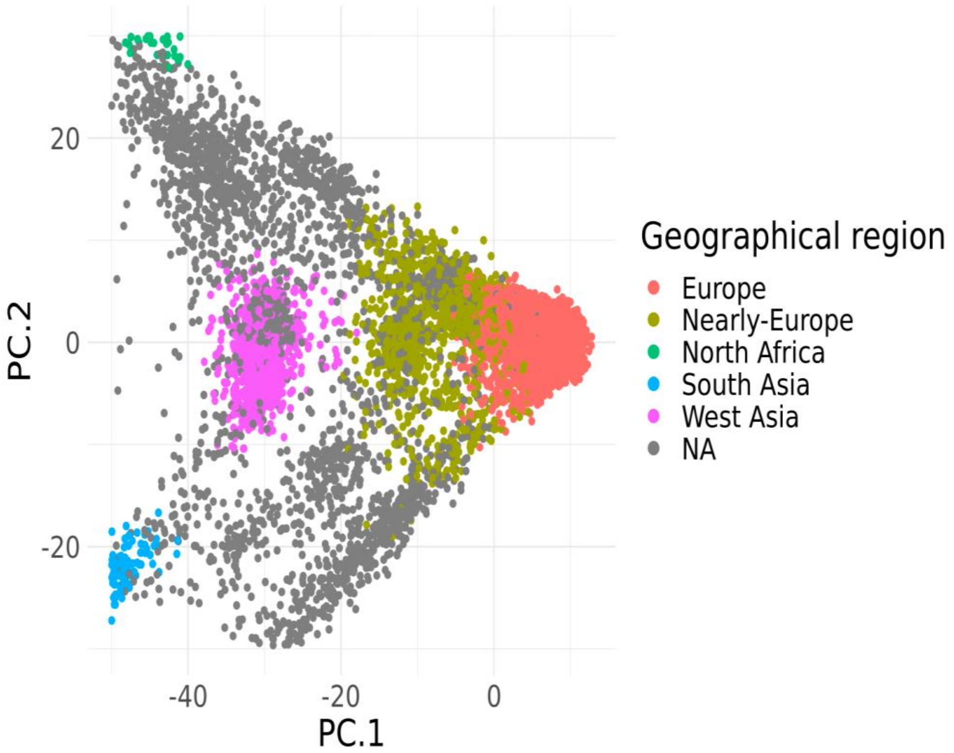

Supplementary Figure 6 Distribution of 25OHD and DBP before and after transformation

Histogram of raw and residualized-ranked inverse normal transformation (RINT) phenotypes for 25OHD (upper panels) and DBP (lower panels). The Y axis shows the counts underlying the distribution of these two variables. In the phenotypes suffixed with res_RINT, the quantification plate effect has previously been regressed out with a linear mixed model, and a rank-inverse normal transformation (RINT) applied to the residuals.

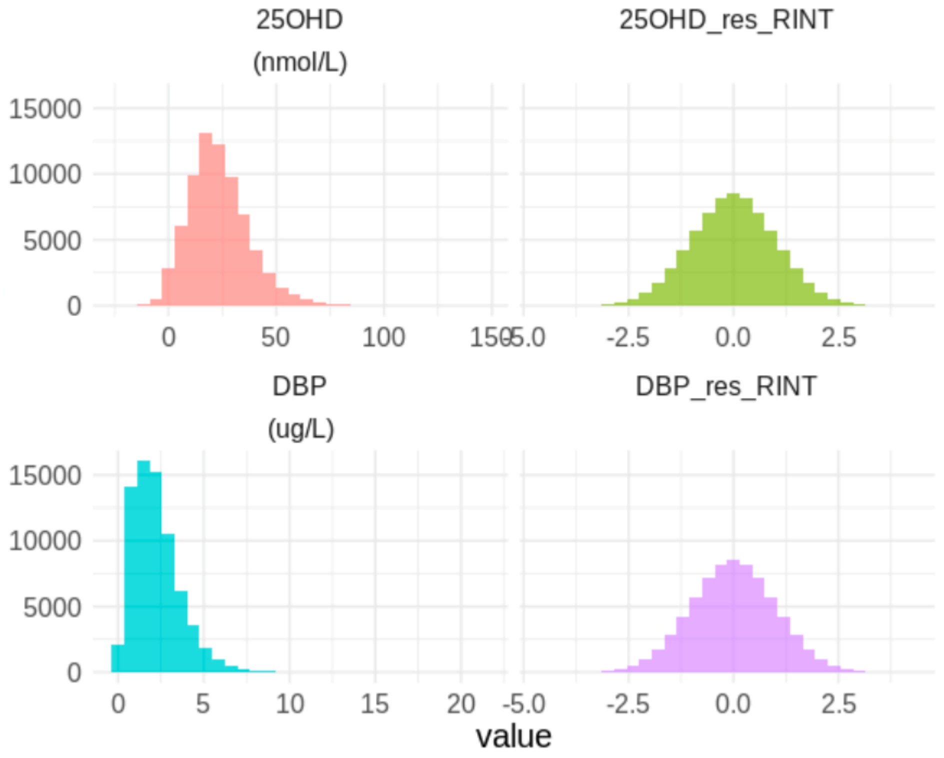

Supplementary Figure 7 Correlation between vitamin D binding protein (DBP and 25-hydroxyvitamin D (25OHD)

Scatter plot of DBP (x axis) and 25OHD (y axis) concentrations in the full sample (Pearson's correlation coefficient r = 0.19, *P*-value < 2.2e-16).

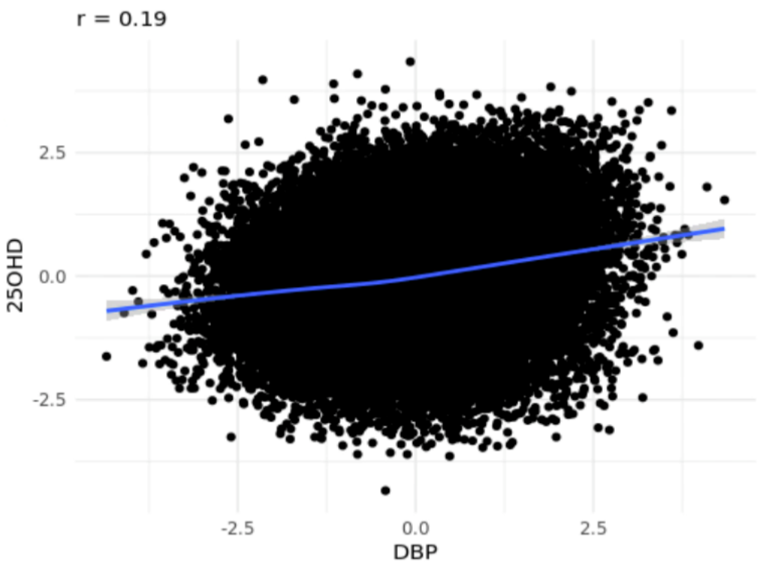

Supplementary Figure 8 Seasonal variation – 25-hydroxyvitamin D (25OHD and vitamin D binding protein (DBP) concentration by month of birth

Mean monthly concentration of 25-hydroxyvitamin D (25OHD) (panel A) and vitamin D binding protein (DBP) (panel B) by month of birth. The error bars show the 95% confidence intervals. The counts for each month (1 to 12) for (a) 25OHD are 5584, 5381, 5498, 5793, 5788, 6192, 6540, 6297, 6525, 6077, 5806, 5731 respectively, and (b) DBP are 5771, 5612, 5912, 5967, 6127, 5724, 6614, 6356, 6374, 5849, 5743, 5895 respectively.

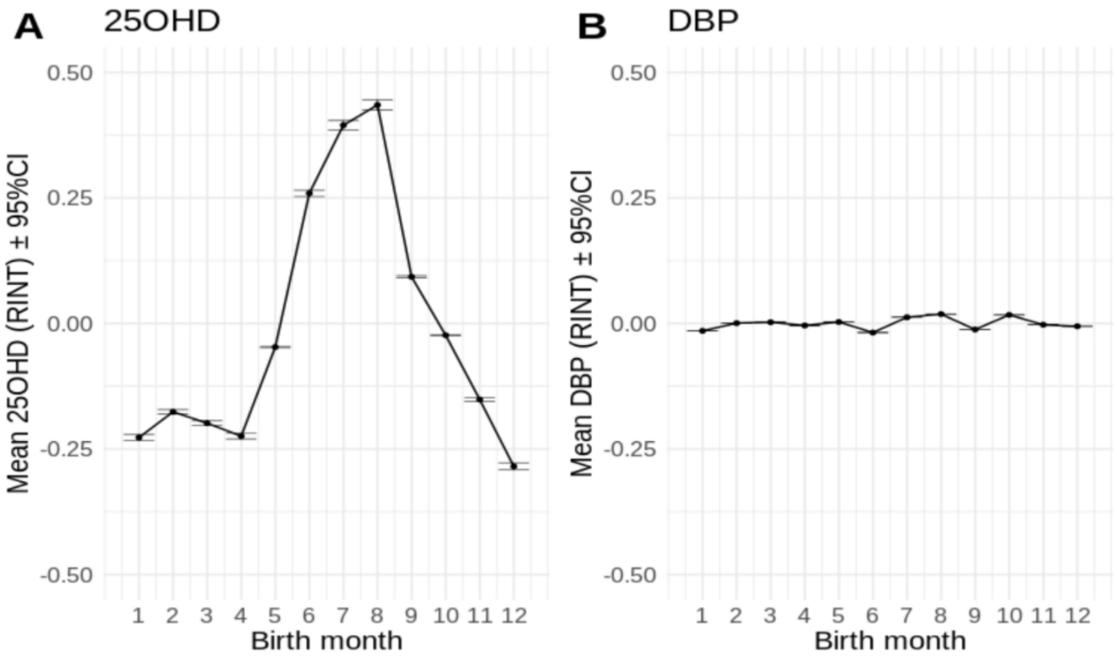

Supplementary Figure 9 DBP and 25OHD concentration by GC haplotypes combinations (diplotypes) in the full sample (European and non-European ancestry)

Distribution of 25-hydroxyvitamin D (25OHD) and vitamin D binding protein (DBP) by the six GC diplotypes in the full sample (European and non-European ancestry). The centre lines show the medians; box limits indicate the 25th and 75th percentiles; whiskers extend 1.5 times the interquartile range from the 25th and 75th percentiles; outliers are represented by dots. The sample sizes for the 1F/1F, 1F/1S, 1F/2, 1S/1S, 1S/2 NS 2/2 diplotypes for (a) 25OHD are 1806, 12429, 5955, 23438, 22229, 5355 respectively; and (b) DBP are 1868, 12549, 6016, 23661, 22419, 5431 respectively.

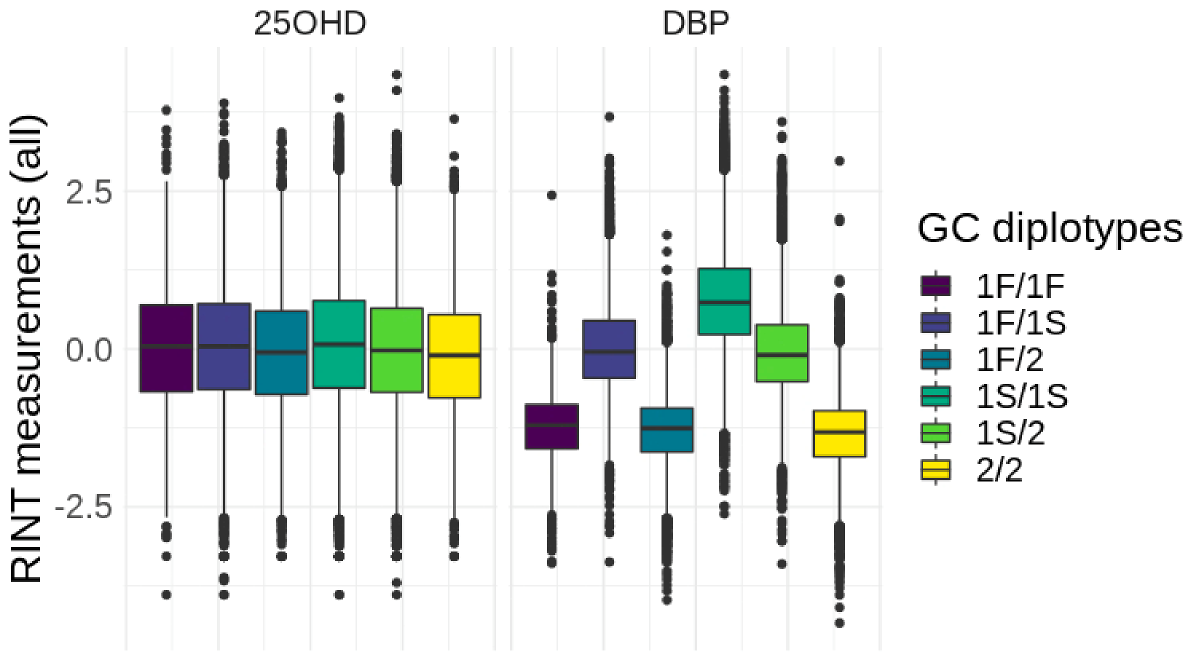

Supplementary Figure 10 GC diplotypes by inferred ancestry groups

Stacked histograms showing the relative proportions of types of GC diplotypes (colour bands) by ancestry-defined subgroups.

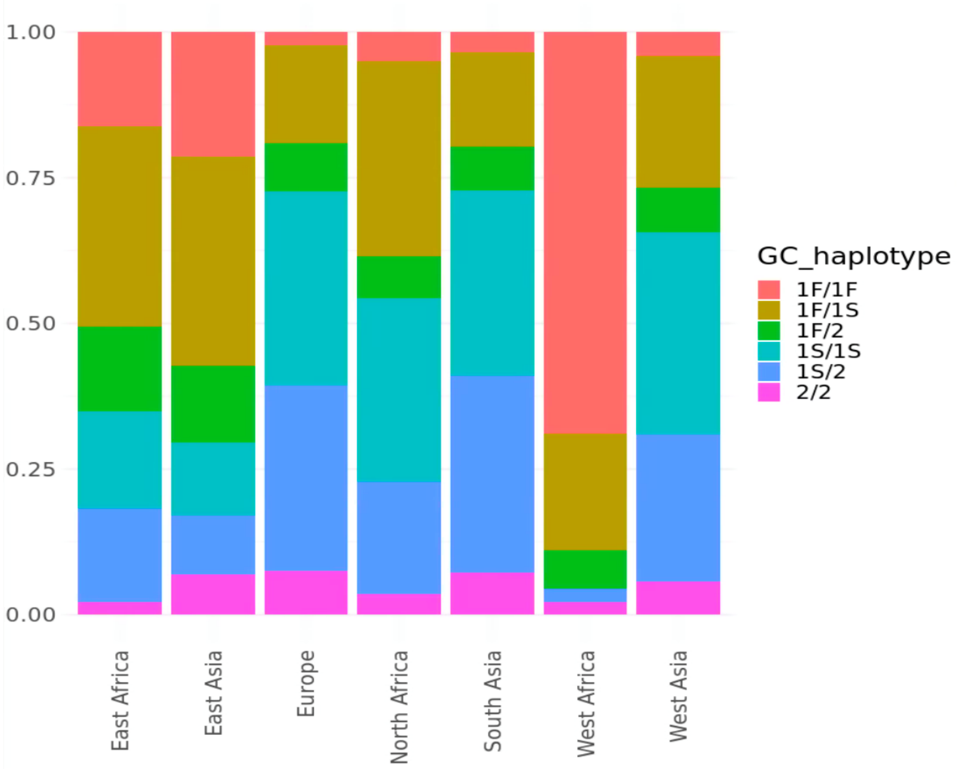

Supplementary Figure 11 Fine mapping of candidate regions – chromosome 4

Fine-mapping results from PolyFun + SuSiE, with corresponding posterior causal probability (PIP) for a 1Mb window around the GC gene, on chromosome 4.

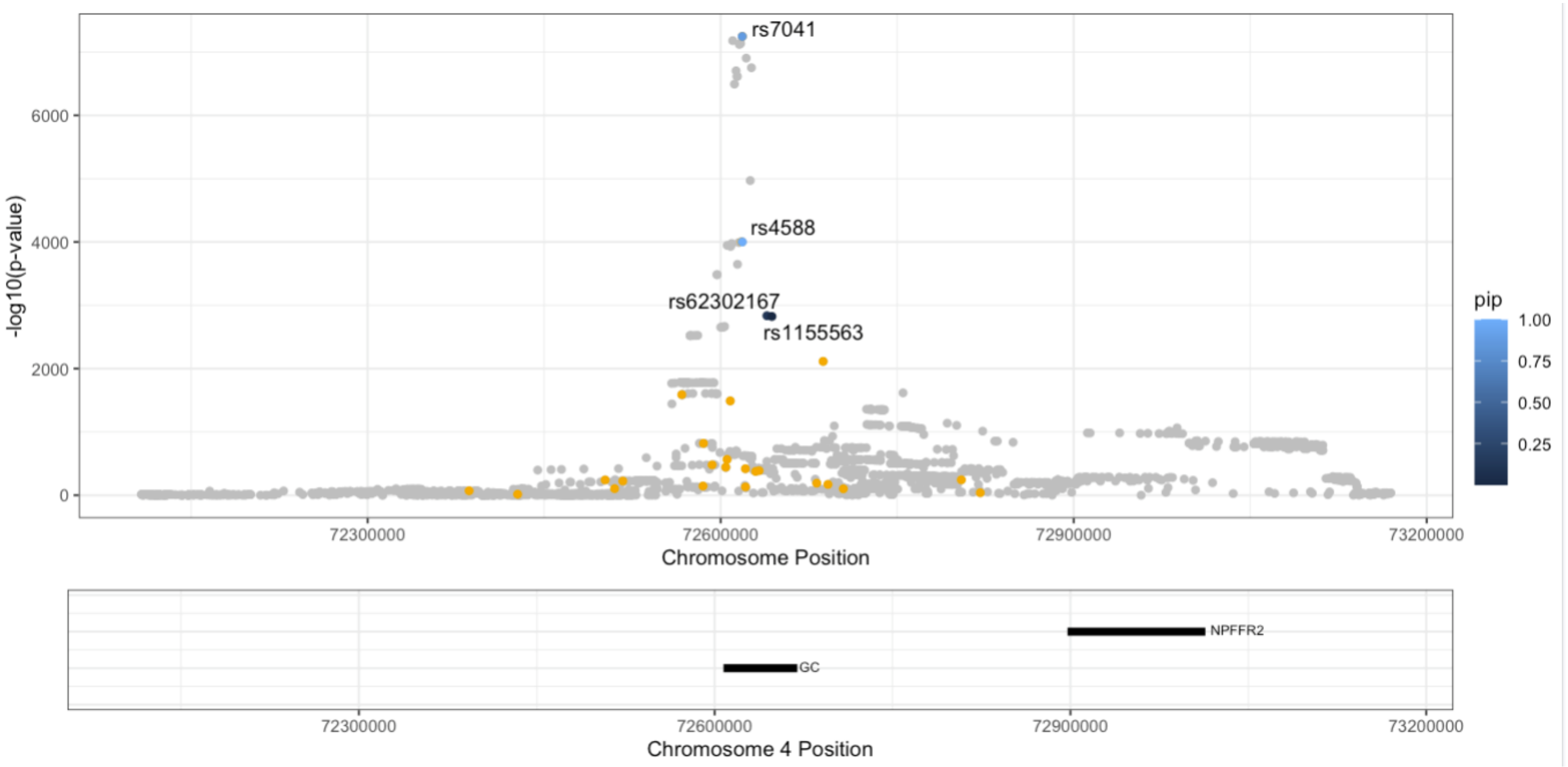

Supplementary Figure 12 Fine-mapping of candidate region – chromosome 12

Fine-mapping results from PolyFun + SuSiE, with corresponding posterior causal probability (PIP) for a 1Mb window around the SH2B3 gene on chromosome 12.

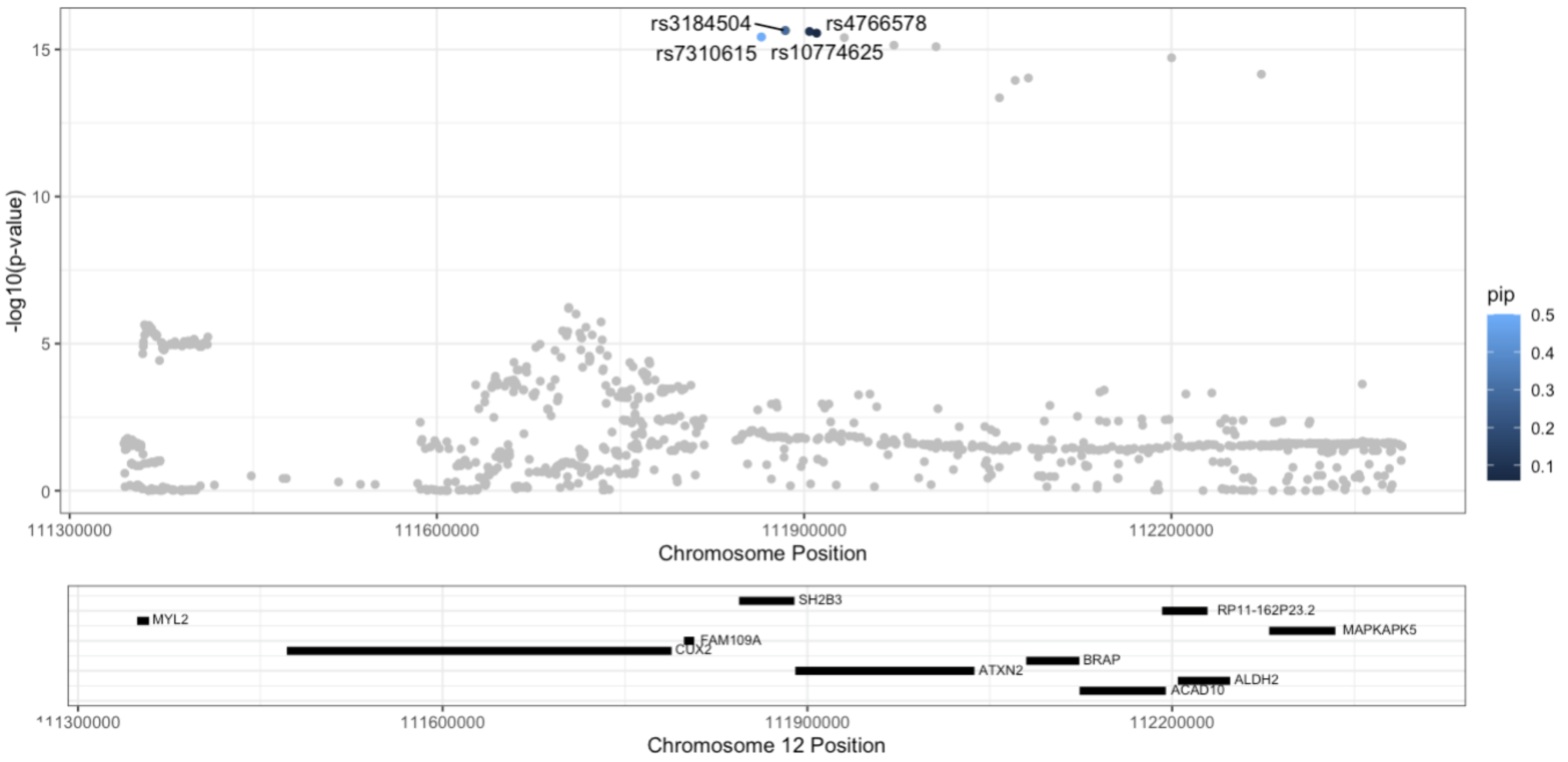

Supplementary Figure 13 Fine-mapping of candidate region – chromosome 17

Fine-mapping results from PolyFun + SuSiE, with corresponding posterior causal probability (PIP) for a 1Mb window around the PSMD3 gene on chromosome 17.

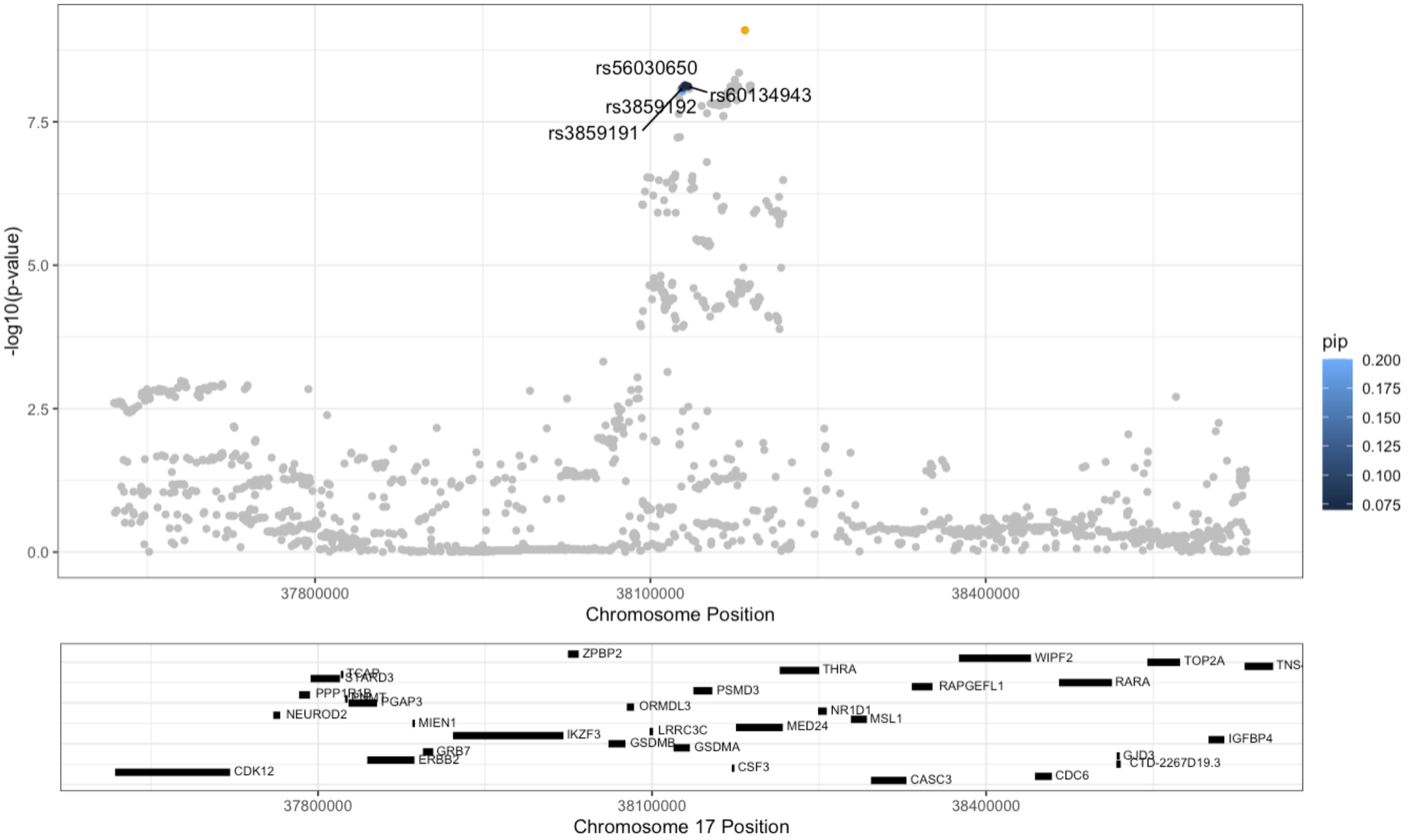

Supplementary Figure 14 Correlation between the SNP estimates for the 25-hydroxyvitamin D (25OHD) GWAS estimates based on the iPSYCH case-cohort versus UK Biobank samples

Scatterplot of the union of the genome-wide significant SNPs (p-value < 5e-8) for the 25OHD GWAS on iPSYCH (x-axis) vs. UKB 25OHD GWAS (y-axis).

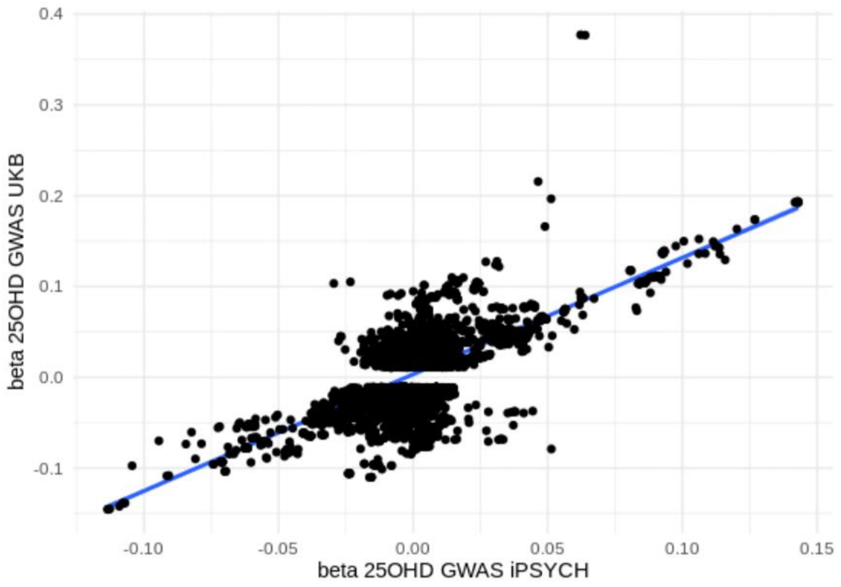

Supplementary Figure 15 Mendelian randomization between GWAS summary statistics for DBP condition on GC (DBP_GC) versus Crohn's disease (CD) and type 1 diabetes (T1D)

GSMR analysis to test for the relationship between DBP conditional on GC and Crohn's disease (CD) and type 1 diabetes (T1D). The GSMR estimates (dashed lines) are accompanied by estimate of effect (beta), standard error and p-value. The GSMR estimate of effect, standard error and p-value were calculated by GSMR. *P*-value was estimated from a two-tailed test. The dash line represents GSMR estimate of effect. The top two panels show the effects of DBP_GC on CD, a) without HEIDI-outlier (SNPs n = 13) and b) with HEIDI-outlier (SNPs n = 11) respectively. We labelled and highlighted the pleiotropic SNPs identified by HEIDI-outlier in orange. Shown in the bottom row are the effects of DBP on T1D, c) without HEIDI-outlier (SNPs n = 13) and d) with HEIDI-outlier (SNPs n = 12). The pleiotropic SNP were labelled and highlighted in red. The error bar shown in the graph represents the GWAS standard errors at each SNP with its centre being the GWAS effect at the SNP. The Bonferroni corrected threshold was 1.92E-3.

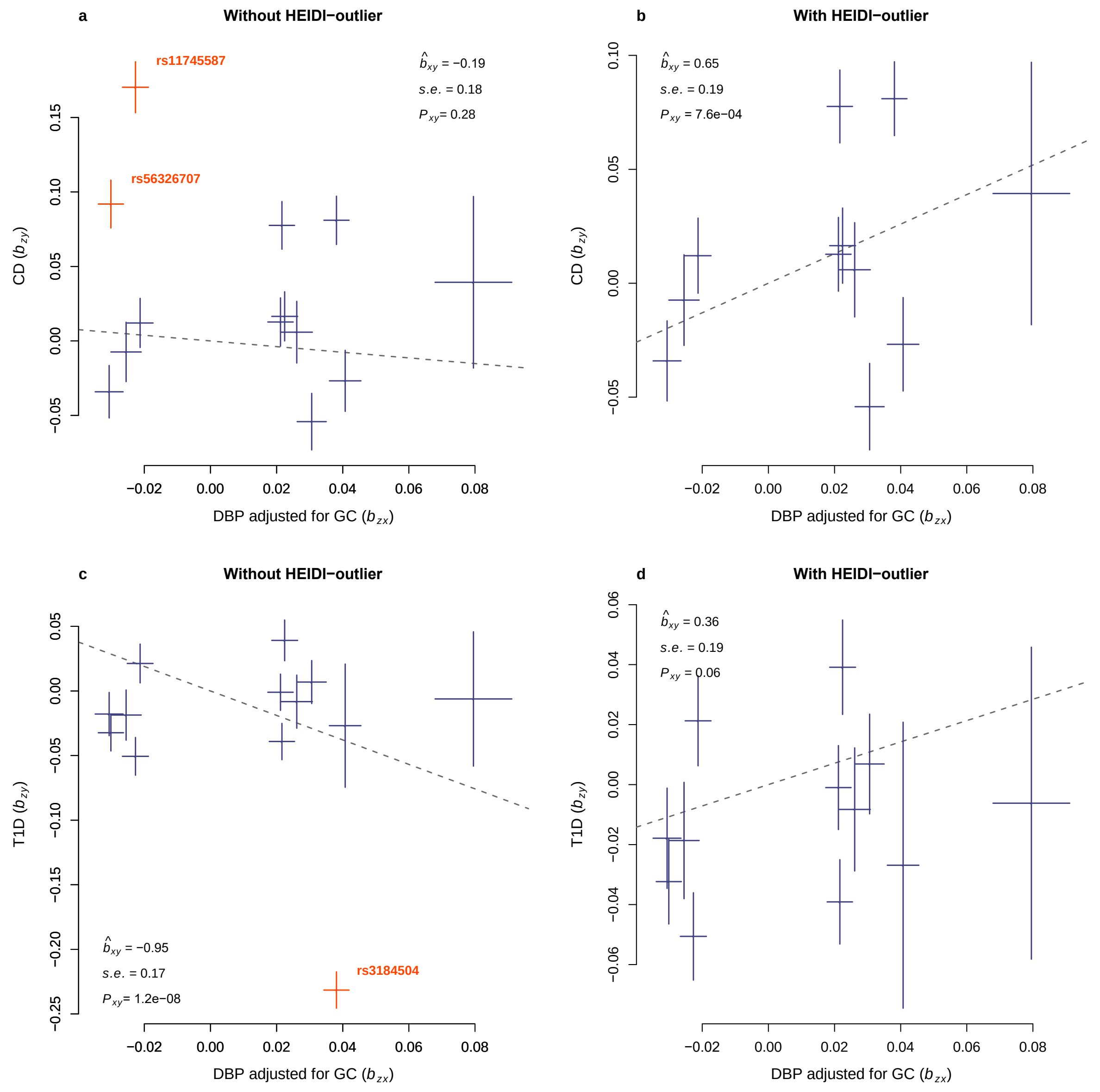

Supplementary Figure 16 Phenome-wide association study (PheWAS) analysis of vitamin D binding protein (DBP) summary statistics in the UK Biobank

PheWAS analysis of DBP concentration in UKB. Shown in the plots are the PheWAS results of DBP. The PheWAS analysis was conducted in UKB with 1,149 phenotypes, 1,027 diseases classified by ICD-10 code, 52 anthropometric and brain imaging measures, 70 infectious disease antigens. The threshold was 4.4×10^{-5}. The significant phenotypes were labelled.

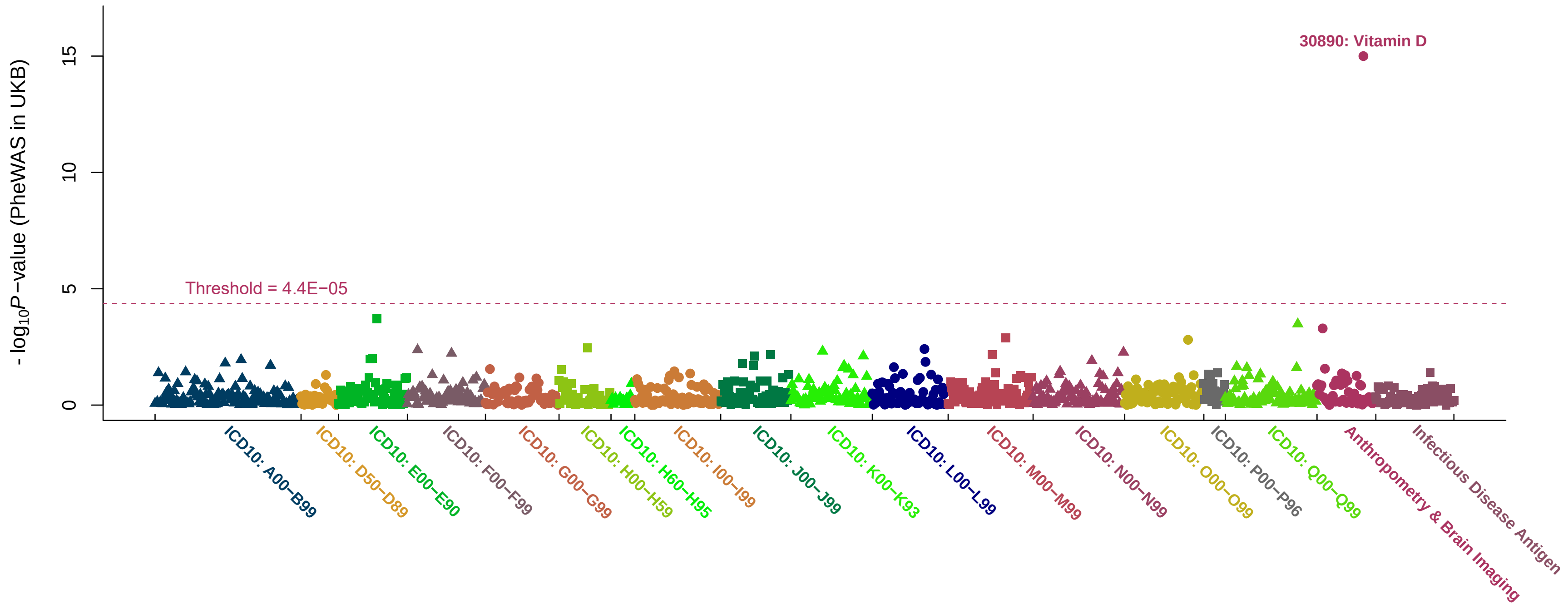

Supplementary Figure 17 Phenome-wide association study (PheWAS) analysis of vitamin D binding protein (DBP) adjusted for GC haplotypes summary statistics in the UK Biobank

PheWAS analysis of DBP concentration in UKB. Shown in the plots are the PheWAS results of DBP adjusted for GC genotypes. The PheWAS analysis was conducted in UKB with 1,149 phenotypes, 1,027 diseases classified by ICD-10 code, 52 anthropometric and brain imaging measures, 70 infectious disease antigens. The threshold was 4.4×10^{-5}. The significant phenotypes were labelled.

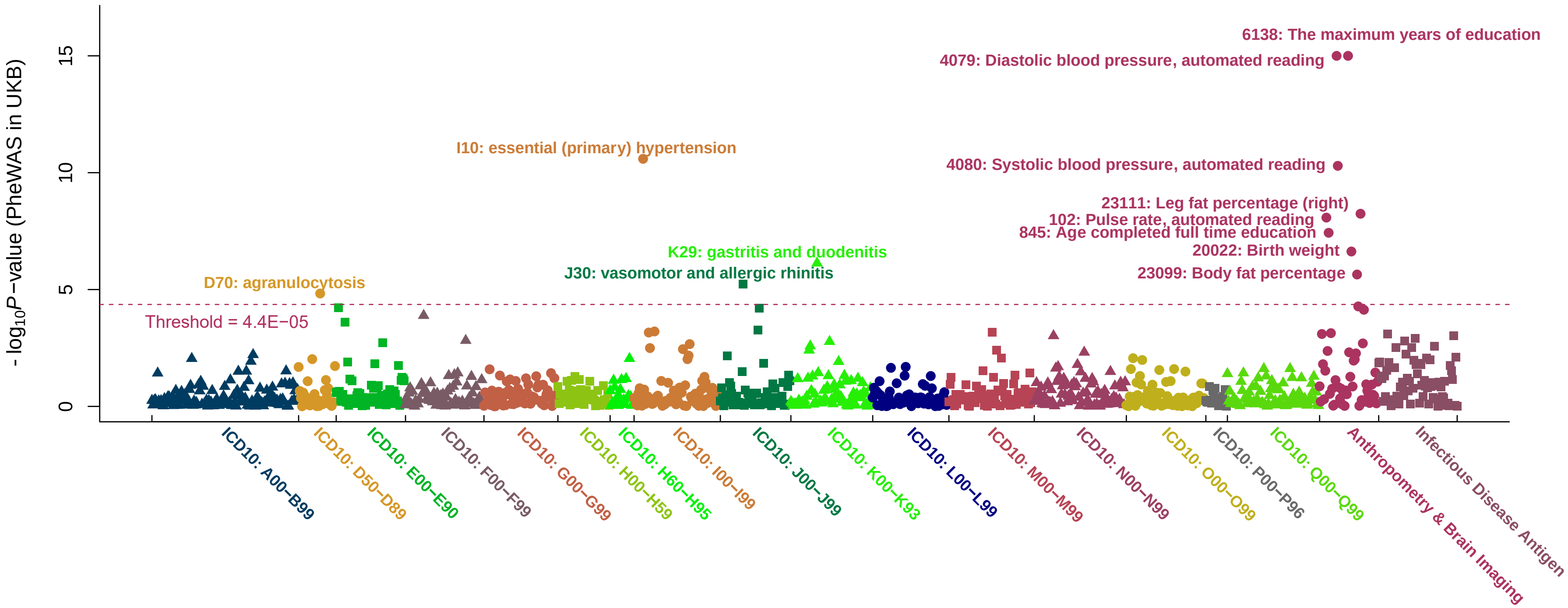

References

1 Zhu, Z. *et al.* Causal associations between risk factors and common diseases inferred from GWAS summary data. *Nat Commun* **9**, 224, doi:10.1038/s41467-017-02317-2 (2018).

2 Revez, J. A. *et al.* Genome-wide association study identifies 143 loci associated with 25 hydroxyvitamin D concentration. *Nat Commun* **11**, 1647, doi:10.1038/s41467-020-15421-7 (2020).
