# Supplementary material for: Genetic correlates of vitamin D-binding protein and 25-hydroxyvitamin D in neonatal dried blood spots
Source: Nat Commun. 2023 Feb 15;14:852. doi: 10.1038/s41467-023-36392-5 (PMC9932173; doi:10.1038/s41467-023-36392-5)
Supplement: Supplementary file 2 — Description of Additional Supplementary Files [file 41467_2023_36392_MOESM2_ESM.pdf]

Description of Additional Supplementary Files

Supplementary Data 1: Covariate associations.

Supplementary Data 2: GWAS SNP-based heritability estimates for 25 hydroxyvitamin D (25OHD), and vitamin D binding protein (DBP) with and without adjustment for GC haplotypes.

Supplementary Data 3: Summary of genome-wide associations estimates for 25 hydroxyvitamin D (25OHD), vitamin D binding protein (DBP) and DBP adjusted for GC (DBP_GC).

Supplementary Data 4: 143 independent associations identified with GCTA-COJO (conditional and joint) on the UKB GWAS and after conditioning on DBP, compared to the iPSYCH 25OHD GWAS

Supplementary Data 5: Supplementary Data 5. Out-of-sample variance explained by the different polygenic scores (prs), adjusted for sex, age and first 20 principal components (PCs).

Supplementary Data 6: Summary of genome-wide associations in the sub-cohort sample only

Supplementary Data 7: FUMA gene-based analysis results

Supplementary Data 8: Results from FUMA gene set analysis

Supplementary Data 9: SMR results of DBP protein concentration

Supplementary Data 10: SMR results of DBP protein concentration conditional on the GC genotypes

Supplementary Data 11: SuSiE fine-mapping results.

Supplementary Data 12: Bi-directional GSMR associations between vitamin D and DBP

Supplementary Data 13: GSMR results of DBP versus selected phenotypes

Supplementary Data 14: GSMR results of DBP adjusted for the GC genotypes versus selected phenotypes

Supplementary Data 15: PheWAS analysis of the DBP concentration in UKB

Supplementary Data 16: Odds ratio of DBP PRS on vitamin D deficiency

Supplementary Data 17: PheWAS analysis of the DBP_GC concentration in UKB
