# Supplementary material for: Genetic correlates of vitamin D-binding protein and 25-hydroxyvitamin D in neonatal dried blood spots
Source: Nat Commun. 2023 Feb 15;14:852. doi: 10.1038/s41467-023-36392-5 (PMC9932173; doi:10.1038/s41467-023-36392-5)
Supplement: Supplementary file 4 — Reporting Summary [file 41467_2023_36392_MOESM4_ESM.pdf]

nature portfolio

Corresponding author(s): John McGrath

Last updated by author(s): Jan 19, 2023

Reporting Summary

Nature Portfolio wishes to improve the reproducibility of the work that we publish. This form provides structure for consistency and transparency in reporting. For further information on Nature Portfolio policies, see our Editorial Policies and the Editorial Policy Checklist.

Statistics

For all statistical analyses, confirm that the following items are present in the figure legend, table legend, main text, or Methods section.

n/a	Confirmed	
☐	☒	The exact sample size (*n*) for each experimental group/condition, given as a discrete number and unit of measurement
☐	☒	A statement on whether measurements were taken from distinct samples or whether the same sample was measured repeatedly
☐	☒	The statistical test(s) used AND whether they are one- or two-sided<br>*Only common tests should be described solely by name; describe more complex techniques in the Methods section.*
☐	☒	A description of all covariates tested
☐	☒	A description of any assumptions or corrections, such as tests of normality and adjustment for multiple comparisons
☐	☒	A full description of the statistical parameters including central tendency (e.g. means) or other basic estimates (e.g. regression coefficient) AND variation (e.g. standard deviation) or associated estimates of uncertainty (e.g. confidence intervals)
☐	☒	For null hypothesis testing, the test statistic (e.g. *F*, *t*, *r*) with confidence intervals, effect sizes, degrees of freedom and *P* value noted<br>*Give P values as exact values whenever suitable.*
☒	☐	For Bayesian analysis, information on the choice of priors and Markov chain Monte Carlo settings
☒	☐	For hierarchical and complex designs, identification of the appropriate level for tests and full reporting of outcomes
☐	☒	Estimates of effect sizes (e.g. Cohen's *d*, Pearson's *r*), indicating how they were calculated

Our web collection on statistics for biologists contains articles on many of the points above.

Software and code

Policy information about availability of computer code

Data collection	The selection of all cases and the random, population-based subcohort for the iPSYCH case-cohort sample was completed by Statistics Denmark and is described elsewhere (PMID: 28924187)
Data analysis	Analyses were conducted over 2021 and 2022 with the most recent versions of the following software packaged: R (versions 4.0.5), MSD Workbench 4.0, the RICOPILI pipeline, many components of the GCTA software package including fastGWA, COJO, GSMR, SMR, mtCOHO, cQTL). LD score regression, SBayes, LDpred2-auto, PolyFun,SuSiE94, Ensembl Variant Effect Predictor (VEP) v85, R packages bigsnpr and bigutilse.

For manuscripts utilizing custom algorithms or software that are central to the research but not yet described in published literature, software must be made available to editors and reviewers. We strongly encourage code deposition in a community repository (e.g. GitHub). See the Nature Portfolio guidelines for submitting code & software for further information.

Data

Policy information about availability of data

All manuscripts must include a data availability statement. This statement should provide the following information, where applicable:

- Accession codes, unique identifiers, or web links for publicly available datasets
- A description of any restrictions on data availability
- For clinical datasets or third party data, please ensure that the statement adheres to our policy

All data generated during this study are included in this published article and its supplementary files. Owing to the sensitive nature of these iPSYCH data (which includes the ANGI subsample), individual level data can be accessed only through secure servers where download of individual level information is prohibited. Each scientific project must be approved before initiation, and approval is granted to a specific Danish research institution. International researchers may gain data access through collaboration with a Danish research institution. More information about getting access to the iPSYCH data can be obtained at https://ipsych.dk/en/about-ipsych.

eQTL data was based GTEX version 8 (the data used for the analyses described in this manuscript were obtained from the GTEx Portal on 02/01/22). Summary statistics from the following studies were used in the GSMR analyses and publicly available: (see related publication for linked references schizophrenia (Ref 101), major depression (102), bipolar disorder (103) , autism spectrum disorder (104), attention deficit hyperactivity disorder (105) , Alzheimer's disease (106), educational attainment (107), multiple sclerosis (108), amyotrophic lateral sclerosis (109), type 1 diabetes (110), Crohn's disease (111), ulcerative colitis (111) , rheumatoid arthritis (112).

We used data from UK Biobank https://www.ukbiobank.ac.uk/ Application Number 12505.

The summary statistics from the GWAS for 25OHD, DBP and DBP adjusted for GC haplotypes are available via the GWAS Catalogue https://www.ebi.ac.uk/gwas/ (Accession numbers GCST90162562, GCST90162563, GCST90162564).

Human research participants

Policy information about studies involving human research participants and Sex and Gender in Research.

Reporting on sex and gender	Sex was included as a covariate in several of the main analyses.
Population characteristics	This study was based on the Lundbeck Foundation Initiative for Integrative Psychiatric Research (iPSYCH) sample, a population-based case-cohort design to study the genetic and environmental factors associated with severe mental disorders. The iPSYCH2012 sample is nested within the entire Danish population born between 1981 and 2005 (n= l,472,762). In total, 86,189 individuals were selected; with 57,377 individuals diagnosed with at least one major mental disorder (schizophrenia, bipolar disorder, depression, autism spectrum disorder (ASD), attention deficit hyperactivity disorder (ADHD)) and a random population cohort of 30,000 individuals sampled from the same birth cohort. By design, there were individuals overlapping between the case sub-cohorts and the random population sub-cohort. We also included 4,791 anorexia nervosa cases (AN; ANG I-DK) from the Anorexia Nervosa Genetics Initiative (ANGI), which has the same design as iPSYCH2012.
Recruitment	All cases were included for the target mental disorders (see above), and in addition, a random population sample of 30,000 was included.
Ethics oversight	The study was approved by the Danish Data Protection Agency, and data access was approved by Statistics Denmark and the Danish Health Data Authority. Approval by the Ethics Committee and written informed consent were not required for register-based projects [Act no. 1338 of 1 September 2020, section 10 on research ethics for administration of health scientific research projects and health data scientific research projects]. All data were de-identified and not recognizable at an individual level.

Note that full information on the approval of the study protocol must also be provided in the manuscript.

Field-specific reporting

Please select the one below that is the best fit for your research. If you are not sure, read the appropriate sections before making your selection.

☒ Life sciences ☐ Behavioural & social sciences ☐ Ecological, evolutionary & environmental sciences

For a reference copy of the document with all sections, see nature.com/documents/nr-reporting-summary-flat.pdf

Life sciences study design

All studies must disclose on these points even when the disclosure is negative.

Sample size	The sample was a population-based case-cohort design (all cases were included) and a random subcohort had a sample size of 30,000. The rationale for the original sample size can be found in a detailed manuscript describing the case-cohort study (PMID: 28924187).

Data exclusions	For the current study, we required the presence of neonatal dried blood samples suitable for analysis of the vitamin D binding protein and 25 hydroxyvitamin D. The entire sample included a total of 86,189 individuals were selected (57,377 individuals diagnosed with at least one major mental disorder). With this sample, 71,944 and 71,212 individuals who had DBP and 25OHD neonatal blood concentrations respectively, 65,694 had data on both.
Replication	Not applicable.
Randomization	Not applicable.
Blinding	All genotyping and neonatal biomarker assays were completed by laboratories who were blind to case status of the samples.

Reporting for specific materials, systems and methods

We require information from authors about some types of materials, experimental systems and methods used in many studies. Here, indicate whether each material, system or method listed is relevant to your study. If you are not sure if a list item applies to your research, read the appropriate section before selecting a response.

Materials & experimental systems

n/a	Involved in the study	
☐	☒	Antibodies
☒	☐	Eukaryotic cell lines
☒	☐	Palaeontology and archaeology
☒	☐	Animals and other organisms
☐	☒	Clinical data
☒	☐	Dual use research of concern

Methods

n/a	Involved in the study	
☒	☐	ChIP-seq
☒	☐	Flow cytometry
☒	☐	MRI-based neuroimaging

Antibodies

Antibodies used	The following antibodies specific for DBP (HYB249-05 and HYB249-01) were supplied by 551 Antibodies (Copenhagen, Denmark).). Extracts were analyzed diluted 1:70 in diluent 101 (#R51AD, MSD). Capture antibodies (used at 10 ug/mL as input concentration) were biotinylated in-house using EZ-Link Sulfo-NHS-LC-Biotin (#21327, Thermo Fisher Scientific) and detection antibodies were SULFO-tagged (R91AO, MSD), both at a challenge ratio of 20:1.
Validation	As a calibrator, we used recombinant human DBP #C953 (Bon Opus, Millburn, NJ, USA). Calibrators were diluted in diluent 101, detection antibodies (used at 1 ug/mL) were diluted in diluent 3 (#R50AP, MSD)

Clinical data

Policy information about clinical studies

All manuscripts should comply with the ICMJE guidelines for publication of clinical research and a completed CONSORT checklist must be included with all submissions.

Clinical trial registration	Not applicable.
Study protocol	The iPSYCH2012 case-cohort sample: new directions for unravelling genetic and environmental architectures of severe mental disorders. PMID 28924187.
Data collection	The iPSYCH2012 sample is nested within the entire Danish population born between 1981 and 2005 (n=l,472,762). In total, 86,189 individuals were selected; with 57,377 individuals diagnosed with at least one major mental disorder.
Outcomes	Concentration of neonatal vitamin D binding protein and 25 hydroxyvitamin D
